# The Functional Consequences of the Novel Ribosomal Pausing Site in SARS-CoV-2 Spike Glycoprotein RNA

**DOI:** 10.3390/ijms22126490

**Published:** 2021-06-17

**Authors:** Olga A. Postnikova, Sheetal Uppal, Weiliang Huang, Maureen A. Kane, Rafael Villasmil, Igor B. Rogozin, Eugenia Poliakov, T. Michael Redmond

**Affiliations:** 1 Laboratory of Retinal Cell & Molecular Biology, National Eye Institute, National Institutes of Health, Bethesda, MD 20892, USA; olga.postnikova@nih.gov (O.A.P.); sheetal.uppal2@nih.gov (S.U.); 2 Department of Pharmaceutical Sciences, School of Pharmacy Mass Spectrometry Center, University of Maryland, Baltimore, MD 21201, USA; whuang@rx.umaryland.edu (W.H.); mkane@rx.umaryland.edu (M.A.K.); 3 Flow Cytometry Core Facility, National Eye Institute, National Institutes of Health, Bethesda, MD 20892, USA; villasmilr@nei.nih.gov; 4 National Center for Biotechnology Information, National Library of Medicine, National Institutes of Health, Bethesda, MD 20894, USA

**Keywords:** ribosome stalling, SARS-CoV-2, spike protein, codon usage, ribosome pausing site

## Abstract

The SARS-CoV-2 Spike glycoprotein (S protein) acquired a unique new 4 amino acid -PRRA- insertion sequence at amino acid residues (aa) 681–684 that forms a new furin cleavage site in S protein as well as several new glycosylation sites. We studied various statistical properties of the -PRRA- insertion at the RNA level (CCUCGGCGGGCA). The nucleotide composition and codon usage of this sequence are different from the rest of the SARS-CoV-2 genome. One of such features is two tandem CGG codons, although the CGG codon is the rarest codon in the SARS-CoV-2 genome. This suggests that the insertion sequence could cause ribosome pausing as the result of these rare codons. Due to population variants, the Nextstrain divergence measure of the CCU codon is extremely large. We cannot exclude that this divergence might affect host immune responses/effectiveness of SARS-CoV-2 vaccines, possibilities awaiting further investigation. Our experimental studies show that the expression level of original RNA sequence “wildtype” spike protein is much lower than for codon-optimized spike protein in all studied cell lines. Interestingly, the original spike sequence produces a higher titer of pseudoviral particles and a higher level of infection. Further mutagenesis experiments suggest that this dual-effect insert, comprised of a combination of overlapping translation pausing and furin sites, has allowed SARS-CoV-2 to infect its new host (human) more readily. This underlines the importance of ribosome pausing to allow efficient regulation of protein expression and also of cotranslational subdomain folding.

## 1. Introduction

Many new and unique features of the novel coronavirus SARS-CoV-2 have contributed to its pathogenicity since its apparent origin in 2019, while the rapid analysis and understanding of the sequence and structure of this virus has been critically important in devising vaccines to thwart the resultant COVID-19 pandemic. An important focus of research has been the surface spike glycoprotein (S protein), which determines virus recognition and endocytosis by human cells [1]. In its evolution, SARS-CoV-2 Spike glycoprotein acquired a new four amino acid -PRRA- insertion sequence at amino acid residues (aa) 681-684. This sequence is absent from all other known lineage B bCoVs such as SARS-CoV and MERS-CoV. This insertion forms a new furin cleavage site in S protein; in addition, there are three new adjacent O-linked glycosylation sites [2]. This is of significance because furin is abundant in the respiratory tract and found throughout the body. Furin cleavage is also used by other formidable RNA viruses, including HIV, influenza, dengue, and Ebola to enter cells [3]. In contrast, the cleavage proteins used by SARS-CoV are much less abundant and widespread, and not as effective. An important question to ask is what are the functional properties of this particular new cleavage site? [3]. Although the virus probably gained the insertion through an as yet unknown illegitimate recombination, this particular furin site sequence architecture has never been found in any other coronavirus from any other species [3].

The functional consequences of the -PRRA- insertion at the protein level seems to be widely accepted and are not debatable. However, it is also well known that the translation of viral RNA depends on various factors. For example, a prominent feature of coronaviruses is the translation frameshift site. Programmed ribosomal frameshifting is an essential mechanism used for the expression of orf1b in coronaviruses [4,5]. Comparative analysis of the frameshift region reveals a universal shift site U_UUA_AAC, followed by a predicted downstream RNA structure in the form of either a pseudoknot or stem loops [4]. It was suggested that programmed ribosomal frameshifting depends on ribosome pausing [6]. It was also suggested that both programmed ribosomal frameshifting and ribosome pausing depend on codon usage biases that are known to be important for efficient RNA translation [7]. Overall, the bodies of evidence from codon usage bias statistics and its associations to tRNA abundance and protein expression offer a compelling narrative for strong translational selection at the RNA level [7,8].

We studied various statistical properties of the -PRRA- insertion at the RNA level (CCUCGGCGGGCA). We determined that the nucleotide composition and codon usage of the insertion sequence are unusual for SARS-CoV-2 genes. This suggested to us that the insertion sequence could cause ribosome pausing. Furthermore, in experimental studies described herein, we demonstrate that the level of expression of “wildtype” original sequence spike protein is much lower than for codon-optimized spike protein in all studied cell lines. However, we were surprised to see that despite this, the original sequence spike sequence produced a higher titer of pseudoviral particles and a higher level of infection. Introduction of a CCAAGG to CCTCGG change (in the first half of the pausing site) and introduction of a full pausing site (CCAAGGAGGGCA to CCTCGGCGGGCA, we used “T” instead of “U”) led to a decrease in the expression of the mutant codon-optimized spike protein, compared to the unmodified fully codon-optimized version. Counterintuitively, the decrease in expression of spike protein in expi293 or LV cells demonstrated a reverse correlation with the amount of pseudoviral particles and the level of infection produced. These findings are discussed in terms of ribosome pausing, slower protein production, proper cotranslational subdomain folding, and successful viral packaging.

## 2. Results

### 2.1. Computational Analysis of the CCTCGGCGGGCA (-PRRA-) Insertion

**Figure 1:**
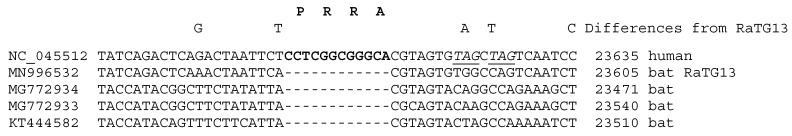
Fragment of the multiple alignments of SARS-CoV-2 S protein RNAs: Sequences surrounding the CCTCGGCGGGCA insertion in the SARS-CoV-2 sequence (GenBank entry NC_045512.2, the SARS-CoV-2 reference sequence). MN996532.1 is the closest bat homolog RaTG13; MG772934.1, MG772933.1, and KT444582.1 are more distantly related bat homologs. Novel out-of-frame stop codons in the human SARS-CoV-2 are italicized.

The novel insertion sequence CCTCGGCGGGCA (unique to the human SARS-CoV-2 genome) is CpG-rich (2 CpG dinucleotides out of 11). There are only 36 nonoverlapping sequences of the same length with two or more CpG dinucleotides in the entire SARS-CoV-2 viral genome (GenBank entry NC_045512.2, 29903 nucleotides), and in bat coronavirus genomes, a much-invoked origin for human SARS-CoV-2 (RaTG13, Figure 1), such CpG-rich regions are even less frequent. Thus, we considered that this human-specific insertion sequence is likely to have unique structural properties.

Indeed, the CGG codon (for arginine) is the rarest codon in SARS-CoV-2 protein-coding genes (Supplementary Table S1). Amongst the 5 least abundant codons, it is found only 9 times, slightly less than the next rarest codon CCC (11 instances) but substantially less than the next rarest codon GCC (found 29 times), while CCT and GCA codons are much more abundant (446 times and 1026 times, respectively). No CGGCGG dicodons, besides the insertion sequence under study, have been found in the SARS-CoV-2 protein-coding genes, making this dicodon an excellent candidate for further functional studies.

One possible hypothesis concerning the functional importance of the insertion sequence is that CGGCGG (and the CCTCGGCGGGCA insertion as a whole) creates a major stumbling block for RNA translation. Such extremely rare dicodons may cause translational pausing and even frameshifting (similar to the programmed translation frameshift in the ORF1ab (SARS-CoV-2 and related viruses). Indirect evidence that a translational frameshift (+1 or +2) could occur is seen in the presence of several SARS-CoV-2-specific out-of-frame stop codons immediately after the pausing site (Figure 1). These stop codons would be expected to suppress extension of the N-end half (S1) of the spike protein in both alternative reading frames as such spurious extensions are likely to produce non-functional proteins. There are no other instances of such neighboring stop codons (as the result of single nucleotide polymorphisms) in the pairwise alignment of the bat RaTG13 CoV genome and the human SARS-CoV-2.

As viral RNA translation occurs in human cells, it is a reasonable expectation that an efficient RNA translation process requires a similar codon usage to the host; this is known for many viruses [9,10,11]. We observed a weak positive correlation between codon usage of SARS-CoV-2 viral genes and all human genes (protein-coding fragments; linear correlation coefficient (CC) = 0.07, Supplementary Table S1). This is consistent with a recent study in which it was shown that codon usage tended to be more similar to that of symptomatic hosts than that of natural hosts, supporting a concept of the general deleterious effect of excessive codon usage similarity between virus and host [12]. Interestingly, the CGG codon is a frequently used codon among arginine codons (AGR, R = A or G; CGN, N = A, T, G or C) in the human protein-coding genes and the rarest one among all codons in SARS-CoV-2 (Supplementary Table S1). For highly expressed ribosomal genes this tendency is weaker (frequency: CGC—24%, AGA—18%, CGG—17%, AGG—15%, CGA—13%, CGT—13%) [13]. It should be noted that most CG-containing codons are somewhat depleted in human protein-coding genes, and almost all CG-containing codons are substantially depleted in SARS-CoV-2 (Supplementary Table S1). This contrasting behavior of CGG codons (0.02% vs. 1.15%) and somewhat similar behavior of the CG-containing codons, in general, in the viral and host genomes remains an open question; various explanations have been put forward [14,15,16,17].

We must emphasize that in this study we are interested in the properties of the insertion sequences [2] rather than in the mechanisms of CpG deficiency in the SARS-CoV-2 genome [14,15,16,17]. Our analysis of human proteins suggests that the -PRRA- sequence, per se, does not have some unexpected statistical properties. We find that the frequency of -PRRA- and shuffled versions of this sequence (e.g., -ARPR-, etc.) is not substantially different: the number of instances of -PRRA- is 375, that of -APRR- is 401, -ARPR- is 307, -PRAR- is 390, and -RPAR- is 285.

Analysis of CCTCGGCGGGCA in human RNA sequences (only protein-coding fragments were analyzed) does not reveal any unexpected properties of this sequence. The CCT-CGG-CGG-GCA stretch of codons is not found in protein-coding regions of human RNA sequences, although the CCTCGGCGGGCA sequence is found 29 times in the strand complementary to the protein-coding strand (antisense strand, Supplementary Table S2). We calculated a similar trend in the pairs of numbers for all possible four codon sequences that encode PRRA (Supplementary Table S2). We built a plot that reflects a potential correlation between the number of codons in the sense and antisense strands, and the CCT-CGG-CGG-GCA sequence is found to have a behavior similar to many other -PRRA-encoding stretches of codons in human protein-coding genes (Supplementary Figure S1 and Table S2).

Analysis of evolutionary conservation including analysis of nonsynonymous and synonymous mutations is a powerful tool to study the functionality of protein-coding regions [18,19]. The estimated number of nonsynonymous and synonymous mutations (single nucleotide variations) in the CCTCGGCGGGCA insertion sequence was obtained from a recent study of mutational patterns in SARS-CoV-2 [20]. The number of nonsynonymous mutations is 10, and the number of synonymous mutations is 5 (Supplementary Table S2C from [20]). The ratio of nonsynonymous vs. synonymous mutations in the CCTCGGCGGGCA insertion sequence is 2. (10/5), whereas the ratio in the 24 base regions (12 to the left and 12 to the right) surrounding the insertion sequence is 3.25 (13/4). This difference is not statistically significant (*p* = 0.699). Thus, the insertion sequence is likely to be under purifying selection (suggesting functional importance) to approximately the same extent as surrounding regions that are known to be evolutionary conserved and are under purifying selection (Figure 1 and [19]) and confirms that this insertion sequence is likely to be functionally important.

Further analyses of mutations using the Nextstrain measure of diversity [21] suggest that the second position of the CCT codon (encoding P681; Figure 1) has an unusually large Nextstrain diversity measure (0.772). It should be noted that a larger value of the diversity measure (0.795) was found for one codon only (the N protein, positions 28883-28885; https://nextstrain.org/ncov/global, accessed date 1 June 2021). The most likely sources of the large diversity measure of the CCT codon are two variants of the -PRRA- insertion sequence (-HRRA- and -LRRA-; Supplementary Table S2C from [20]). The genetic diversity measure of other positions of the insertion sequence (Figure 1) varies between 0 and 0.03, suggesting strong purifying selection. Codon usage frequencies of CAT and CTT codons (corresponding to -HRRA- and -LRRA- variants) are not dramatically different from the CCT codon (Supplementary Table S1).

All these observations suggest that the insertion sequence may have some important functional properties at the RNA level that are likely to be associated with RNA translation. Thus, this putative association warranted further experimental studies.

### 2.2. Description of Spike Protein Constructs

Based on the bioinformatic analyses, and to compare the expression of the original sequence spike protein and codon-optimized spike protein, we obtained the codon-optimized S protein cDNA construct (CCAAGGAGGGCA furin site, pCMV-codon-optimized spike, VG40589-UT, Sino Biologicals) and the original sequence cDNA construct (CCTCGGCGGGCA furin site, pUNO1-SARS2-S, InvivoGen). Additionally, we constructed the pSelect-nCoV-S_green fluorescent protein (GFP) construct cloning original sequence full spike cDNA from (pUC57-2019-nCoV-S (Original), MC_0101080, Genscript) into pSELECT-GFP-Zeo vector. To study the effect of the putative pausing site in codon-optimized spike protein, we performed site-directed mutagenesis to change three nucleotides in two stages in the furin site and constructed the three mutants: QC11 (CCAAGGCGGGCA reconstructing the second CGG in the furin site; modified position underlined), QC22 (CCTCGGAGGGCA reconstructing the first CGG in the furin site), and QC24 (CCTCGGCGGGCA, reconstructing all original codons in the furin site) to study the role of the pausing site and its effect on the expression of S protein.

### 2.3. Expression of Spike Protein (Various Constructs) in Different Cell Lines

**Figure 2:**
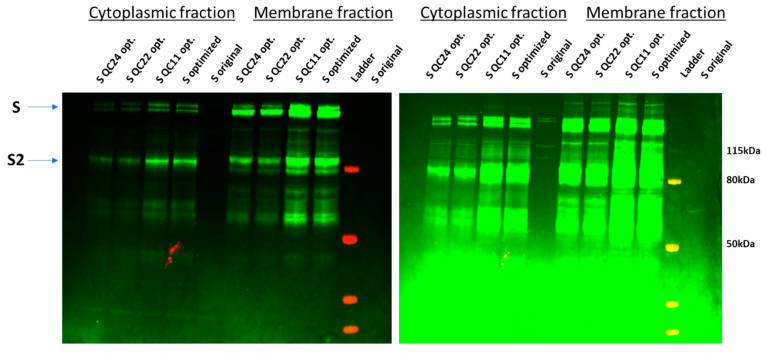
Expression of original and codon-optimized S proteins in HEK293F cell lysates. The left panel was imaged at normal gain, while the right panel was imaged at higher gain to visualize original S protein expression (S original). Experiments were repeated several times.

All five constructs were expressed in HEK293F cells. We found that all four codon-optimized full-length spike (S) proteins, as well as the S2 fragments, were produced, as confirmed by immunoblotting and mass spectrometry analysis (Figure 2 and Supplementary Table S3). The original SARS-CoV-2 spike protein (pUNO1-SARS2-S) was produced in much lower amounts and was observable by immunoblotting only at a higher gain setting on the far-red imager (Figure 2) and was not detectable by mass spectrometry analysis. Incorporation of the first half of the predicted pausing site or the full pausing site led to a significant drop in S protein expression (Figure 2). A similar pattern was held for Expi293 and LV cells (modified HEK293 cells for lentiviral production of original vs. codon-optimized S protein; Supplementary Figure S2).

### 2.4. The Effect of the Novel Predicted Pausing Site on Expression of SARS-CoV-2 Spike Glycoprotein Variants in Lentiviral Pseudotypes

**Figure 3:**
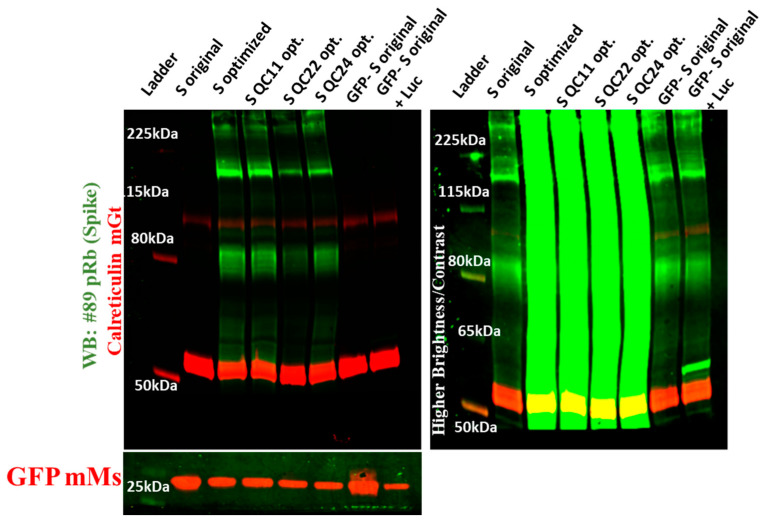
Expression of original and codon-optimized S proteins in Expi293 cell lysates. The left panel was imaged at normal gain, while the right panel was imaged at higher gain to visualize original S protein expression (S original). Experiments were repeated several times.

To study the differences in expression of SARS-CoV-2 S protein variants with and without the predicted pausing site, we produced lentiviral pseudovirions with SARS-CoV-2 S-containing envelope constructs using psPAX2 (packaging) and pLenti-GFP (transfer) constructs. For the pSelect-nCoV-S_GFP envelope construct, we employed the same packaging construct but a different transfer construct (pLenti-Luc) to avoid any cross-reactivity with GFP. We utilized three cell lines for lentiviral pseudovirion production: Expi293, LV cells, and HEK293T cells. All three are derivatives of the HEK293 cell line: the first is optimized for recombinant protein expression, the second is optimized for production of lentiviral particles, and the third is an adherent HEK293 subtype. We collected supernatants 48 h after transfection and concentrated the pseudovirions and the cell pellets to determine the level of expressed S protein trapped in the cells and incorporated in pseudovirions. We observed the same pattern of expression in cell pellets of Expi293 cells after triple transfection and collection of pseudovirions (Figure 3). Original sequence spike protein produced alone or simultaneously with GFP protein was expressed in much lower amounts compared to codon-optimized spike protein constructs. Additionally, inserting the first half or both halves of the pausing site led to a decrease in protein expression.

**Figure 4:**
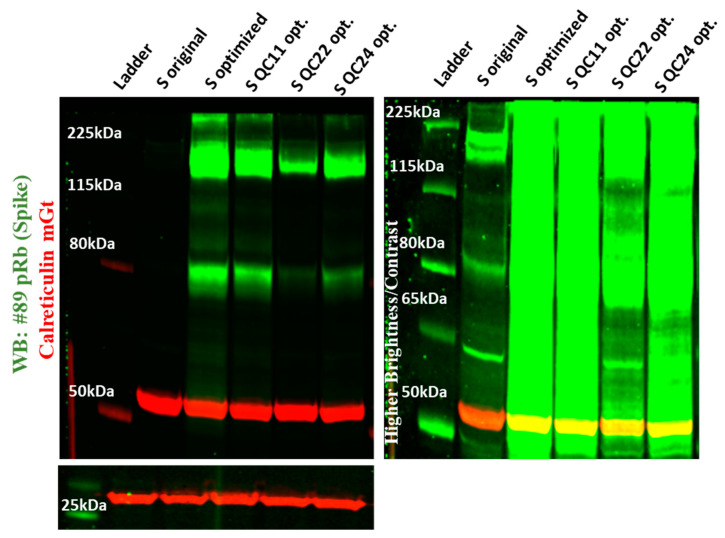
Expression of original and codon-optimized S proteins in HEK293T cell lysates. The left panel was imaged at normal gain, while the right panel was imaged at higher gain to visualize original S protein expression (S original). Experiments were repeated several times.

We observed the same pattern of expression in cell pellets of HEK293T cells after triple transfection and collection of pseudovirions, where the original wildtype S protein was produced in low amounts compared to codon-optimized S protein, which was produced in the highest amount. Mutants incorporating corrections of the half or full pausing (furin) site in codon-optimized S protein produced in intermediate amounts (Figure 4).

**Figure 5:**
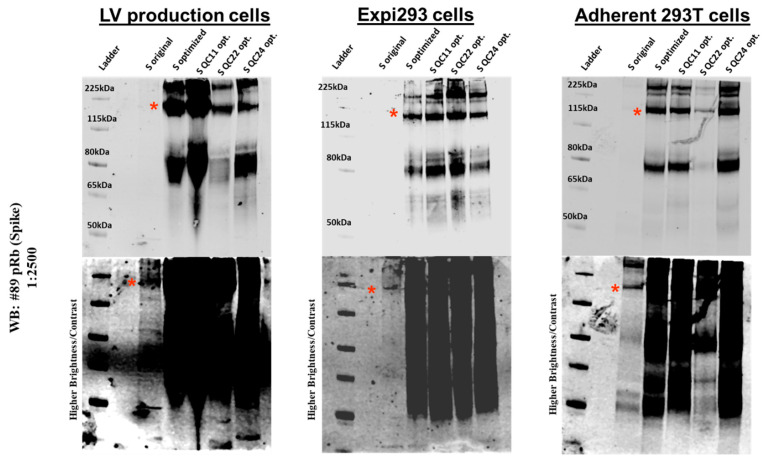
Expression of S protein in pseudovirions expressing original and codon-optimized S proteins. Pseudovirions were produced in LV production cells, Expi293 cells, and adherent HEK293T cells. Upper panels were imaged at normal gain, while lower panels were imaged at higher gain to visualize original S protein expression (S original). Red asterisk indicates spike protein band.

To determine the SARS-CoV-2 S protein (original and wildtype or mutant-optimized) levels in viral particles produced in different cell lines, viral supernatants were concentrated, and the viral particles resuspended in 30 μL of RIPA buffer, and 5 μg of total protein was separated by SDS-PAGE and probed by an antibody against full-length S protein. We again observed a low level of original S protein expression and a much higher level of expression of codon-optimized S protein with some decrease of expression for mutants containing partial or whole pausing site (Figure 5).

### 2.5. Infection of Various Cells with Spike Protein Variant Pseudotyped Particles

**Figure 6:**
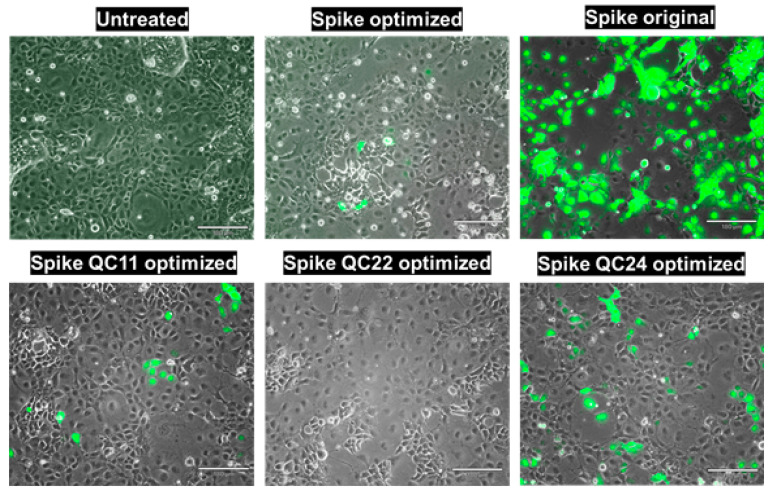
COS7 infection with LV-produced pseudovirions. Experiments were conducted in duplicate and repeated several times. Scale bar = 180 μm.

**Figure 7:**
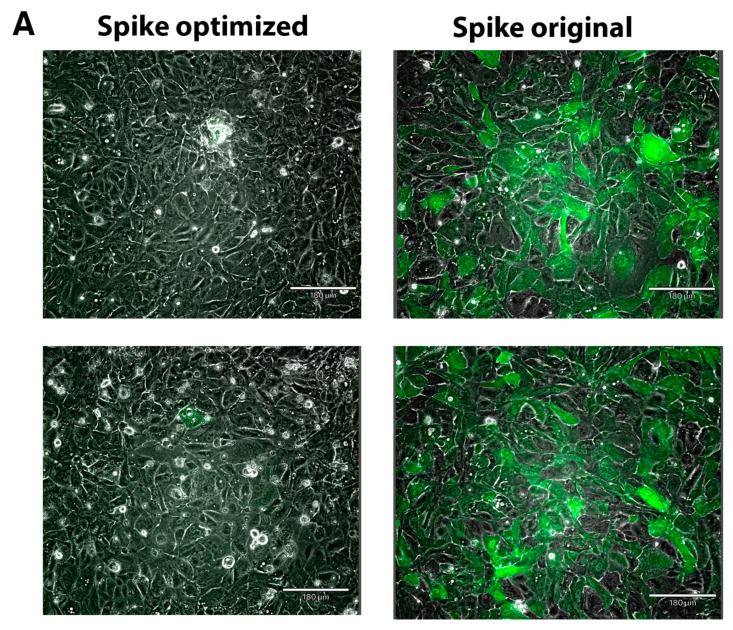

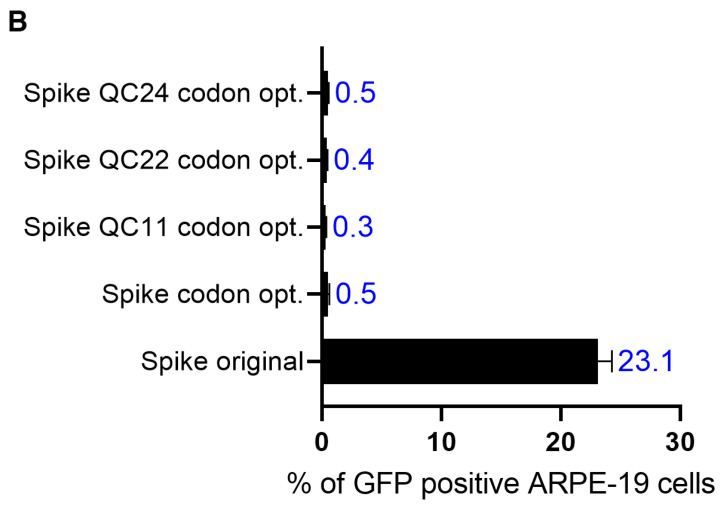
Infection of ARPE-19 cells with Expi293 produced pseudovirions: (**A**) GFP fluorescence analysis. Experiments were conducted in duplicate and repeated several times; scale bar = 180 μm; (**B**) %GFP-positive cells measured by Cytation7. Error bars indicate the standard deviation.

We infected COS7, ARPE-19, and African green monkey kidney epithelial Vero E6 cells with spike protein pseudotyped particles. In all cases the same ratio of plasmid DNA (Spike (envelope) along with psPAX2 (packaging) and pLenti-GFP (transfer) was transfected to LV, Expi293, or HEK293T cells. We used the same amount of spike plasmid DNA for all constructs according to recommendations for transfection in each cell line. Interestingly, for all three production cell types, the pseudovirion titer was much higher for the original spike protein construct, compared to the optimized construct. The titer was measured based on qPCR and p24 capsid protein quantitation. We started with LV-produced pseudovirions and infected COS7 cells. Even though we started with exactly the same amount of the different spike DNA constructs, we observed 609-fold more original sequence spike-containing pseudovirions than the codon-optimized one but only 27-fold more than the QC24 mutant of codon-optimized S protein (incorporating the reintroduced pausing site), measured by qPCR of WPRE and LTR (Supplementary Figure S3). Only the original sequence S protein-containing pseudovirions and, to a lesser degree, the QC24 mutant successfully infected COS7 cells (Figure 6). As the difference in the production of pseudovirions is so large in LV cells, we next decided to produce them in Expi293, where we found the differences in S protein production for original sequence and codon-optimized variants to be less. Again, we started with the same amount of S protein cDNA constructs and collected the pseudovirions from cell-conditioned medium supernatant. When we infected ARPE-19 cells, we again observed much higher infection rates for original sequence S protein, compared to the codon-optimized construct, along with partial recovery of infection for the QC24 full pausing site codon-optimized mutant (Figure 7 and Supplementary Figure S4).

**Figure 8:**
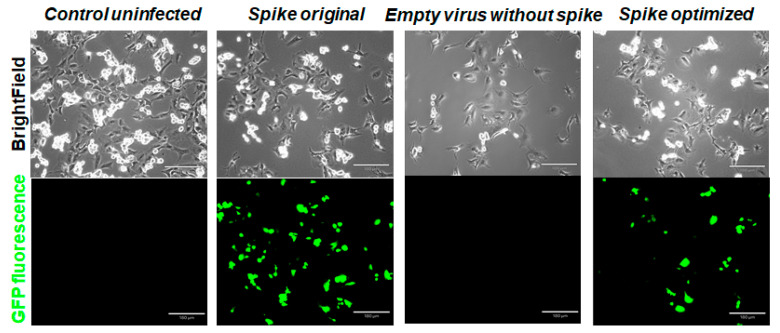
Vero E6 infection with HEK293T-produced pseudovirions measured by GFP fluorescence. Experiments were conducted in duplicate and repeated several times. Scale bar = 180 μm.

**Figure 9:**
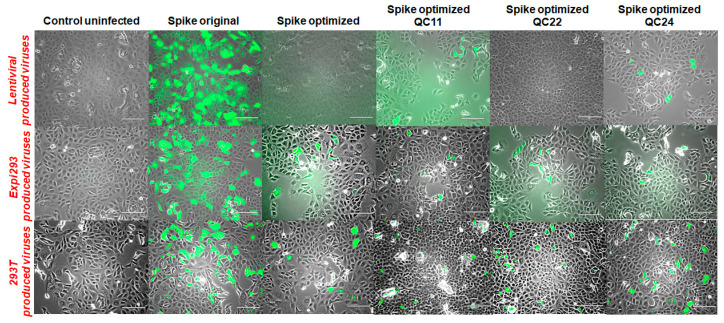
Vero E6 infection with LV-, Expi293-, and HEK293T-produced pseudovirions measured by GFP fluorescence in infected cells. Experiments were conducted in duplicate and repeated several times. Scale bar = 180 μm.

**Figure 10:**
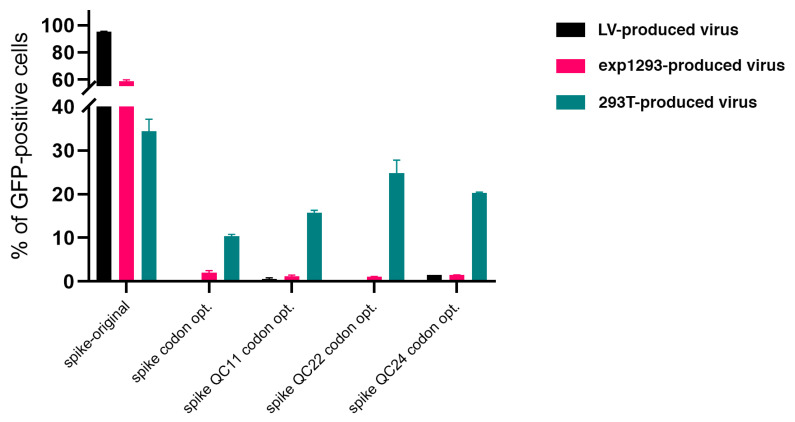
Quantification of GFP expression by cytometry in Vero E6 cells following lentiviral infection. Error bars indicate the standard deviation.

Next, we decided to compare infection of the Vero E6 monkey cell line with pseudotyped lentiviral particles produced in all three types of production cells. First, we confirmed that transformation with empty vector without spike protein, along with packaging and transfer constructs, does not produce virus, and target cells do not contract an infection. Additionally, we confirmed that transfecting HEK293T cells with the same ratio of codon-optimized spike construct, along with packaging and transfer constructs, produce a much lower level of infection, compared to the original sequence spike construct (Figure 8). Similar results were obtained with Expi293-produced virus constructs (Supplementary Figure S5). Then, we compared the infection rates of Vero E6 cells with pseudovirions produced in all three production cell lines (Figure 9). We measured the number of GFP-positive cells in each sample and observed a higher rate of infection for the original sequence spike pseudovirions vs. codon-optimized ones. With the reintroduction of the pausing site, the level of infection was restored to some degree, as seen by the number and percentage of GFP-positive cells (Figure 10). When we measured LTR and WRPE qPCR and p24 levels (to measure the functional titer of spike pseudotyped lentiviral particles), we observed much higher titer levels for original sequence spike pseudovirions than codon-optimized ones. Titer rose with the reintroduction of half or of the complete pausing site (Supplementary Table S4). For all cell types, LTR qPCR, WRPE qPCR, and p24 pseudovirion titers tightly correlate to %GFP-positive infected cells (Table 1). Thus, the smaller was the amount of spike protein produced in the cells and incorporated in the pseudovirions, the more lentiviral pseudotyped particles were produced, and the greater infection of host cells was observed.

## 3. Discussion

In this study, we analyze the functional consequences of a dual-effect insert comprised of a unique ribosomal pausing site in the SARS-CoV-2 spike glycoprotein RNA that encodes an equally unique polybasic furin cleavage site. The latter (furin site) is well known as an important aspect of SARS-CoV-2 infectivity; however, the former (ribosomal pausing site) may be equally important as we show herein that it plays an important role in modulating the expression level of the spike protein. This would appear to regulate appropriate expression of properly infective spike protein in infected host cells. While we do not directly address the evolutionary origins of this crucially important insertion, a topic of great interest, our findings do shed light on how the SARS-CoV-2 virus, via this insertion, has adapted to its new human host and also been an agent of morbidity and mortality.

Recently, translation elongation has emerged as an important contributor to the regulation of protein expression at the mRNA level, beyond transcriptional and translation initiation events [22]. There are numerous translation quality control checkpoints for the successful production of mature proteins and associating them correctly with their interaction partners [22]. Specifically, ribosomes pause to allow cotranslational protein folding [23,24,25], protein targeting, or protein interactions, and this pausing is dictated by a combination of the mRNA sequence (e.g., presence of stretches of rare codons), along with the variability of different tRNA concentrations [22].

However, ribosome pausing can also lead to ribosome collisions and cotranslational degradation of both mRNA and the nascent protein chain [26,27,28,29]. Thus, there is a complex interplay between the positive and negative effects of ribosome pausing [26,27]. In general, the amount of tRNA that recognizes rare codons is lower, and the elongation rate correlates well with the total tRNA pool, suggesting that the charging of tRNAs is not rate limiting [30]. It is presumed that rare codons and dicodons overrepresented in the first 90–100 nucleotides of open reading frames of all kingdoms of life and often found at boundaries of protein domains, slow translation elongation to help in the folding of proteins [30,31,32]. Consequently, ribosome pausing is likely to be required for successful protein folding [30]. In this study, we observed that codon-optimization of SARS-CoV-2 spike protein for protein expression does not optimize either lentiviral pseudovirion production or infection. Reintroduction of the rare codon putative pausing site in the codon-optimized spike construct led to less protein expression but higher titers of packaged lentiviral pseudovirions and higher levels of infection of host cells. Therefore, we suggest that a rare-codon putative pausing site may be important for slower protein production, proper cotranslational subdomain folding, and successful viral packaging of SARS-CoV-2 virions [24,25]. Considering that CGG is the rarest codon in the SARS-CoV-2 genome, the CCT-CGG-CGG-GCA stretch of codons is likely to be beneficial for pausing of translation and the efficient folding of the two-domain S protein in the context of local tRNA pools that have been attenuated by viral translation [33,34]. This is consistent with the observation that CCT-CGG-CGG-GCA stretch of codons is not found in protein-coding regions of human RNA sequences (Supplementary Table S2), although CCT, CGG, and GCA are frequently used codons in human protein-coding genes (Supplementary Table S1). Therefore, the CCT-CGG-CGG-GCA stretch of codons is expected to have somewhat unusual behavior during translation of the S protein in human cells.

It is also well known that the translation of viral RNA depends on various factors [4,5,6]. For example, a prominent feature of coronaviruses is the translation frameshift site [4]. Programmed ribosomal frameshifting is an essential mechanism used for the expression regulation of orf1b in coronaviruses [4,5]. It has been suggested that programmed ribosomal frameshifting depends on ribosome pausing [6]. Evidence that a translational frameshift (+2 or +3) is possible due to ribosome stalling in the -PRRA- sequence is the emergence of two SARS-CoV-2-specific out-of-frame stop codons immediately after the pausing site (Figure 1). These stop codons would be expected to suppress the extension of the N-end half of the spike protein in both alternative reading frames. There are no other instances of such neighboring stop codons. Thus, the emergence of these stop codons may be associated with the -PRRA- insertion. Our mass spectrometry and immunoblot analyses confirmed S2 presence in the original sequence, codon-optimized, and mutant codon-optimized expressed proteins, and we did not observe any change in the pattern of S protein expression in all the studied constructs. It should be noted that the mass spectrometry analysis of S protein was performed in conditions that may not reflect all stages of native SARS-CoV-2 and it that is likely to be much more complex, compared to what we know about the details of virus transcription, translation, and replication processes. For example, an unexpected 24 bases deletion in the S protein has been detected in a substantial fraction of subgenomic viral RNAs [35]. Deep sequencing and ribosomal profiling data showed that the fraction of this genomic deletion is small (~2%) at the early stages of viral infection. However, this fraction increases at late stages of infection; therefore, it probably does not substantially affect viral transcripts and translation of S protein [36].

While we restricted our study to the original Wuhan isolate S protein to establish the underlying mechanism, analyses of mutations using the Nextstrain measure of diversity [21] suggested that the second position of the CCT codon (encoding P681, Figure 1) has an unusually large Nextstrain diversity measure. The most likely source of the large diversity measure of the CCT codon are two variants of the -PRRA- insertion sequence (-HRRA- and -LRRA-; Supplementary Table S2C from [20]). An extremely large diversity value may be the result of positive selection similar to other positions in the S protein [37]. The genetic diversity measure of other positions of the insertion sequence (Figure 1) varies between 0 and 0.03, suggesting strong purifying selection acting toward functional conservation of nonsynonymous and synonymous sites. This is consistent with the suggested functional importance of the insertion sequence at the RNA level. The functional significance of -HRRA- and -LRRA- variants is not clear [38,39] although it was suggested that the -HRRA- that is present in the B.1.1.7 strain (the “UK Strain”) is likely to impact infection and pathogenesis of the virus [40]. This remains a controversial issue because even the overall impact of the furin protease in infectivity was recently questioned [38,39]. Our results suggest that the insertion sequence is likely to function as a translation pausing site at the RNA level. Thus, this functional property of the insertion sequence may have a similar (or even greater) impact, compared to furin site functional properties at the protein level. Codon usage frequencies of CAT and CTT codons (corresponding to -HRRA- and -LRRA- variants) are somewhat similar to the CCT codon (Supplementary Table S1); thus, we do not expect that these variants substantially attenuate translation of the insertion sequence, although it cannot be excluded that observed differences may cause substantial changes in properties of the translation pausing [4,5,6]. It is a possibility that the observed large divergence of -(P/L/H)RRA- and functional properties of these variants are somehow associated with host immune responses and the effectiveness of SARS-CoV-2 vaccines (https://asm.org/Articles/2021/February/SARS-CoV-2-Variants-vs-Vaccines, accessed date 28 May 2021), although this possibility clearly awaits further investigation.

Another possibility that should be taken into consideration is that the reduced pseudovirus formation/infectivity of the overexpressed optimized spike protein sequence could be due, at least in part, to its overwhelming host–cell post-translational processing mechanisms (such as glycosylation). For example, we cannot exclude a possibility of a protein glycosylation bottleneck, which might be essential for a stable S protein maturation. In this regard, it has been demonstrated that Sindbis virus infection of Chinese hamster ovary cells substantially altered the protein glycosylation processes of infected cells, with both abnormal truncated oligosaccharides and normal full-sized oligosaccharides being transferred from lipid-linked precursors to newly synthesized viral glycoproteins [41]. Furthermore, glucose starvation leads to a severe under glycosylation of viral glycoproteins, with some glycosylation sites not acquiring covalently linked oligosaccharides [42]. The spike protein is heavily glycosylated at both N- and O-linked sites, and impaired glycosylation would clearly affect the proper maturation of spike protein and concomitant virus/pseudovirus formation [43]. Conversely, glycosylation of the three new O-linked glycosylation sites adjacent to the polybasic furin site [2] decreases furin cleavage of S protein [44]. These findings underscore the critical importance of S protein glycosylation but do not detract from our overall conclusion that the novel ribosomal pausing site encoding the furin site acts as a brake on translation to allow proper modulation of spike protein cotranslational folding that is absent in the overexpressed optimized spike protein sequence.

Synonymous mutations leading to changes in codon and dicodon usage may be associated with some hereditary diseases [45,46]. For example, McCarthy et al. [47] examined 35 synonymous single nucleotide polymorphisms linked to disease and proposed that dicodon usage, instead of codon usage, could be responsible for altered translational kinetics and monogenic hereditary diseases. In addition to monogenic hereditary diseases, some complex diseases (for example, autistic spectrum disorders, ASD) are associated with translation pausing and codon usage changes [48,49,50,51,52]. Furthermore, it has been recently suggested that a promising approach for viral vaccine development is to generate an attenuated virus through codon pair deoptimization. This approach only requires limited knowledge specific to the virus in question, other than its genome sequence [53]. Therefore, it is well suited for emerging viruses (including SARS-CoV-2), for which we may not have extensive data [53]. However, these techniques require caution because we cannot always predict how optimization and deoptimization will affect translation as well as folding and packaging of the coronavirus and how well codon-optimized or deoptimized version of the protein will correspond to native antigen.

While the complete translational value of our findings is unclear at this point in time, they support the possibility that antiviral drugs that promote amino acid starvation/synthesis by reducing fractions of rare codon tRNAs could be useful in blocking viral protein translation. One such example of a drug targeting protein translation is the prolyl-tRNA synthetase inhibitor halofuginone [54], which has been shown recently to be a potent inhibitor of SARS-CoV-2 infection and replication [55]. Further in-depth study of such drugs is clearly warranted.

In conclusion, it is likely that this insertion of overlapping translation pausing and furin sites has allowed SARS-CoV-2 to infect its new human host more readily. This underlines the importance of the punctuated mode of evolution [56,57], at least for viruses. It is also consistent with the recent finding of the novel SARS-CoV-2 strain with numerous substitutions (including a mutation giving rise to a -HRRA- site instead of the -PRRA- site) and a deletion. Most likely, these changes occurred during a short period of time (https://virological.org/t/preliminary-genomic-characterisation-of-an-emergent-sars-cov-2-lineage-in-the-uk-defined-by-a-novel-set-of-spike-mutations/563, access date 3 June 2021). Similar bursts of novelty due to insertion sequences may be a general property of the evolution of coronaviruses [58].

## 4. Materials and Methods

### 4.1. Computational Analysis

The original Wuhan isolate (GenBank entry NC_045512.2) was used as the reference SARS-CoV-2 genome. Multiple alignments of viral genomes were reconstructed using the MUSCLE program [59]. Mutational data were obtained from Supplementary Table S2C in reference [20]. We used mutations that were observed more than once to avoid sequencing errors. The presence/absence of multiple mutations was used to study modes of evolution. A comparison of mutations (nonsynonymous and synonymous) was performed using the two-tailed Fisher’s exact test (https://www.graphpad.com/quickcalcs/contingency1.cfm; access date 27 March 2021). Further analyses considering frequencies of mutations were performed using the Nextstrain measure of diversity [21]. Codon usage of human protein-coding genes was obtained from the Codon Usage Database (https://www.kazusa.or.jp/codon/; access date 9 November 2020).

### 4.2. Constructs and Plasmids

Original sequence SARS-CoV-2 spike protein (GenBank NC_045512.2) was amplified from puno1-SARS2-S plasmid (InvivoGen, San Diego, CA, USA) and cloned into pSelect gfp plasmid (InvivoGen) BamH1 using Gibson assembly (GA) with the following primers: sprot_fwdgfp: agatcaccggcgtgtcgacgATGTTTGTTTTTCTTGTTTTATTGC and sprot_revgfp: cccatggctgcagagcgctgTTATGTGTAATGTAATTTGACTCCTTTG.

For construct stability, we used NEB stable cells according to manufacturing protocol with small changes (transformation outgrowth performed at room temperature for 2 days. Routine *E. coli* cultures were grown at 30 °C).

The control pCMV-VSV-G plasmid, lentiviral packaging, and transfer plasmid psPAX2, plenti-CMV-gfp, and pLenti-CMV-luc were obtained from Addgene (Watertown, MA, USA).

### 4.3. Cell Lines

SARS-CoV-2 spike protein was transiently produced in HEK293, Expi293, and LV-MAX suspension cells. Cells were grown according to the manufacturer’s instructions (Thermo Fisher Scientific, Waltham, MA, USA). Production of SARS-CoV-2 pseudovirions was carried out in LV-MAX suspension cells with LV-MAX™ Production Medium, Expi293F™ suspension cells with Expi293F™ Expression Medium and HEK293T adherent cell line in DMEM, with 10% FBS supplementation. Pseudovirions were used to infect COS7, ARPE-19, and African green monkey kidney cell line Vero E6 (#CRL-1586; ATCC, Manassas, VA, USA). Vero E6 cells were maintained in DMEM media with 10% FBS supplementation.

### 4.4. Production of SARS-CoV-2 S Protein Pseudovirions

Pseudovirions were produced by cotransfection of cells with packaging plasmid psPAX2, pLenti-GFP transfer plasmid, and plasmids encoding either SARS-CoV-2 S protein (original sequence or optimized with mutations), VSV-G, or empty vector. Pseudovirion production was slightly modified according to the cell line used, e.g., the amount of total DNA and transfection reagent used.

For lentiviral production cells (LV-MAX suspension, cat #A35684, Thermo Fisher Scientific) we followed the manufacturer’s protocol. We used the optimized ratio 3:2:2 for Package:Transfer:Spike plasmids; a total of 70 mg/30 mL culture. Media was collected at 48h post transfection (Table 2).

For Expi293F™ cells (cat #A14527, Thermo Fisher Scientific) cells, we followed the manufacturer’s protocol with the same ratio of plasmids (3:2:2) with a total of 30 mg DNA/30 mL cell culture using 293fectin™ Transfection Reagent (Thermo Fisher Scientific) (Table 2).

For HEK 293T adherent cells we used the same ratio of plasmids (3:2:2) with a total of 30 µg per 2 × 10^6^ cells for transfection using FuGENE^®^ 6 Transfection Reagent (Promega Corporation, Madison, WI, USA). Media supernatant was collected at 48h post transfection (Table 2).

The supernatants were harvested at 48 h post transfection and centrifuged at 800× *g* for 5 min and passed through a 0.45 μm filter. Pseudotyped virus stocks were aliquoted and stored in cryovials at −80 °C.

### 4.5. Measurement of Physical and Infectious Viral Titer

Vero E6 (African green monkey kidney cell line) cells were seeded at a cell density of 1 × 10^5^ in 6-well plates and infected with 400 µL of pseudotyped virus. GFP fluorescence in the infected cells was visualized using a Revolve microscope (Discover Echo, San Diego, CA, USA) using a 10x objective. At 48 and 72 h after transduction, the percentages of GFP-positive cells were measured. Cell cytometry was performed in the original cell culture plate using a Cytation instrument (Model CYT7UW, BioTek, Winooski, VT, USA). All cell numbers were per well and were identified using a high contract mask on brightfield images. GFP-positive cells were identified using a mask on fluorescent images using Gen5 Image Prime Software Version 3.10 (BioTek).

To measure physical titer, we used p24 and qPCR assays. RNA was extracted using Maxwell RCS Viral TNA (Promega AS1330) followed by turbo DNAse treatment (Invitrogen AM1907). cDNA was synthetized (High-Capacity cDNA Reverse Transcription Kit, Thermo Fisher Scientific) and used for TaqMan qPCR with primers (listed in Table 3) for LTR and WPRE [60] (Table 3). Standard curves were obtained using pLenti plasmid.

Lentiviral p24 protein levels were measured using Lenti-X™ GoStix™ Plus (TaKaRa, Mountain View, CA, USA). As per the manufacturer’s protocol, 20 µL of lentiviral cell supernatant was applied to the cassette and incubated for 10 min at room temperature. The band that appears was scanned using a smartphone camera or equivalent mobile device running the GoStix Plus app. The latter calculated the viral titer (ng/mL p24) by comparing the intensities of the test and control bands.

### 4.6. Immunoblot Analysis of S Proteins

Expi293 and HEK 293T cells transfected with lentiviral plasmids were harvested after 48h post transfection by centrifuging at 1500× *g* for 10 min at room temperature. The cell pellets were lysed using RIPA buffer (20 mM Tris-HCl pH 7.5, 150 mM NaCl, 1 mM EDTA, 0.1% SDS, 1% NP-40, and 1× protease inhibitor cocktail), sonicated and centrifuged at 16,000× *g* for 10 min to clear the nuclear debris, and the supernatant (total cell lysate) was collected in a clear tube. Protein estimation was performed using Pierce™ Coomassie Plus (Bradford) Assay Kit (Thermo Fisher Scientific). Samples were prepared in 4× SDS loading dye and heated at 95 °C for 10 min. Then, 30 μg of total protein was loaded on Invitrogen 8% and 4–12% Bolt Plus Bis-Tris gels (Thermo Fisher Scientific) and subjected to immunoblotting for spike protein (1:3000 dilution, SARS-CoV-2 Spike Antibody Rabbit pAb; Cat # 40589-T62; Sino Biologicals Wayne, PA, USA), calreticulin (1:2000 dilution, Goat mAb; Abcam, Cambridge, MA, USA), GFP (1:2500 dilution, mouse mAb; Cell Signaling Technology, Danvers, MA, USA) and secondary antibodies (1:15000 dilution) in Intercept™ Blocking buffer (LI-COR Biosciences, Lincoln, NE, USA). Membranes were scanned on an Odyssey Infrared Imager (LI-COR Biosciences) and image data were processed using Image Studio™ Lite V3.1 (LI-COR Biosciences).

For assessing S protein expression in pseudoviral particles, lentiviral supernatant was concentrated using Lenti-X-Concentrator solution (Takara Bio USA, Inc., Mountain View, CA, USA) in a 3:1 ratio, as per the manufacturer’s protocol. The mixed solution was incubated at 4 °C for 30–90 min, followed by centrifugation at 1500× *g* at 4 °C for 45 min. The concentrated pellet of viral particles was resuspended in RIPA lysis buffer and protein estimation was performed using Pierce™ Coomassie Plus (Bradford) Assay Kit. Samples were prepared in 4× SDS loading dye and heated at 95 °C for 10 min. Additionally, 5 μg of total protein was loaded on 8% Bolt Plus Bis-Tris gel and subjected to immunoblotting to detect S protein.

### 4.7. Mass Spectrometry Analysis

Gel bands with molecular weights corresponding to spike protein and its variants were excised from Invitrogen 8% and 4–12% Bolt Plus Bis-Tris gels and cut into 1 × 1 mm pieces. The gel pieces were destained in 50% acetonitrile with 50 mM ammonium bicarbonate. Proteins in the gel pieces were reduced by 50 mM Tris(2-carboxyethyl)phosphine and alkylated by 100 mM iodoacetamide in the dark. The gel pieces were dehydrated by neat acetonitrile and air dried. Proteins were digested and peptides were extracted by incubating the gel pieces with sequencing grade trypsin in 50 mM ammonium bicarbonate overnight. Tryptic peptides from the supernatant were desalted and separated on a nanoACQUITY UPLC analytical column (BEH130 C18, 1.7 μm, 75 μm × 200 mm, Waters; Milford, MA, USA) over a 165-min linear acetonitrile gradient (3–40%) with 0.1% formic acid on a Waters nano-ACQUITY UPLC system and analyzed on a coupled Thermo Scientific Orbitrap Fusion Lumos Tribrid mass spectrometer, as described previously [61]. Full scans were acquired at a resolution of 240,000, and precursors were selected for fragmentation by collision-induced dissociation (normalized collision energy at 35%) for a maximum 3 s cycle. Tandem mass spectra were searched against the UniProt reference protein sequence of SARS-CoV-2 spike glycoprotein and the reference *Homo sapiens* proteome of the expression host using Sequest HT algorithm [62] and MS Amanda algorithm [63] with a maximum precursor mass error tolerance of 10 ppm. Carbamidomethylation of cysteine and deamidation of asparagine and glutamine were treated as static and dynamic modifications, respectively. The resulting hits were validated at a maximum false discovery rate of 0.01 using Percolator, a semi-supervised machine learning algorithm [64].

## Figures and Tables

**Table 1:** Correlation between qPCR and p24 results for the constructs and %GFP-containing infected cells.

48 Hours	LV Cells	Expi 293 Cells	HEK293T Cells
qPCR (WPRE)	0.99961	0.99951	0.87737
qPCR (LTR)	0.99866	0.99578	0.55399
p24	0.99995	0.98458	0.64395

**Table 2:** Total amount DNA used for transfection and recommended by manufacturer.

Cell Line	Total DNA µg	Media mL	Transfection Reagent
HEK293T	30	30	FuGENE 6^®^
LV-MAX	70	30	LV-MAX
Expi293F	30	30	293fectin™

**Table 3:** Primers for qPCR of LTR and WPRE.

NAME	SEQUENCE 5′-3′
LTR-fw	TGTGTGCCCGTCTGTTGTGT
LTR-rev	GAGTCCTGCGTCGAGAGAGC
LTR-probe	5′-FAM-CAGTGGCGCCCGAACAGGGA-TAMRA-3
WPRE-fw	CCGTTGTCAGGCAACGTG
WPRE-rev	AGCTGACAGGTGGTGGCAAT
WPRE-probe	5′-FAM- TGCTGACGCAACCCCCACTGGT-TAMRA-3

## Data Availability

The data presented in this study are available in this article and Supplementary Materials here.

## Supplementary Materials

The following are available online at https://www.mdpi.com/article/10.3390/ijms22126490/s1.
