# Supplementary material for: The Functional Consequences of the Novel Ribosomal Pausing Site in SARS-CoV-2 Spike Glycoprotein RNA
Source: Int J Mol Sci. 2021 Jun 17;22(12):6490. doi: 10.3390/ijms22126490 (PMC8235447; doi:10.3390/ijms22126490)
Supplement: Supplementary file 1 [file ijms-22-06490-s001.zip › ijms-1236394-supplementary.pdf]

Supplementary Figures and Tables

Figure S1. Comparison of the incidence of the CCT-CGG-CGG-GCA stretch of codons and other sequences synonymously encoding -PRRA- in sense and antisense strands of protein-coding human sequences (see Supplementary Table S2 for tabulation of sequence incidence).

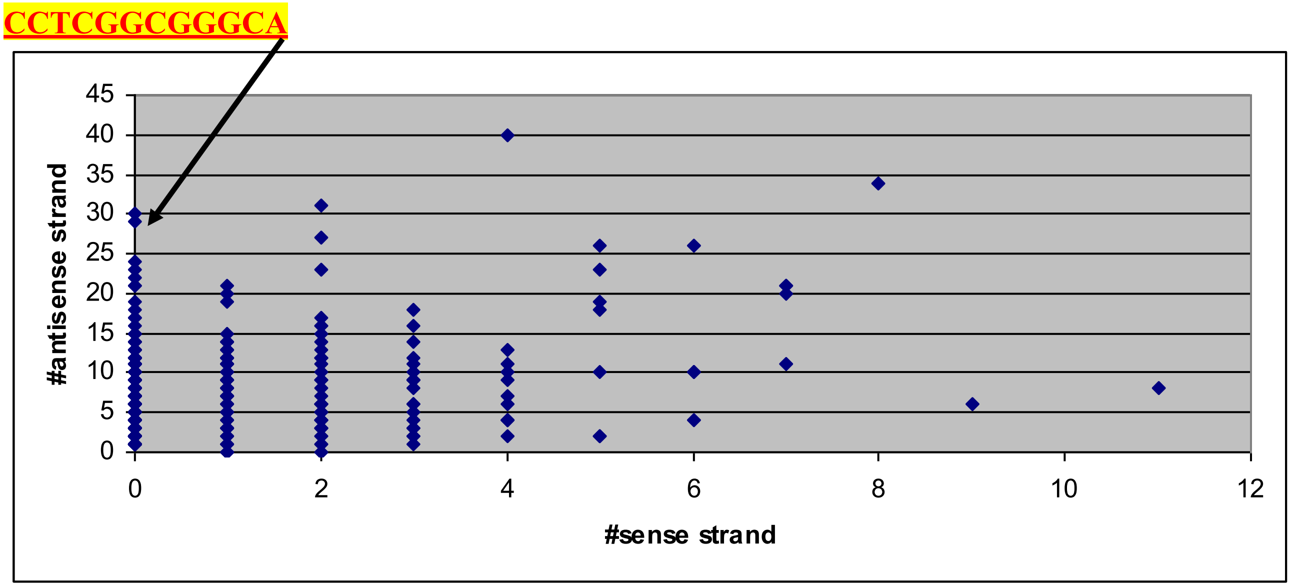

Figure S2. Expression of original and codon-optimized S proteins in LV cell lysates (A) and in Expi293 cell lysates (B). In (A), left panel was imaged at normal gain, while right panel was imaged at higher gain to visualize original S protein expression (S original). Experiments were repeated several times.

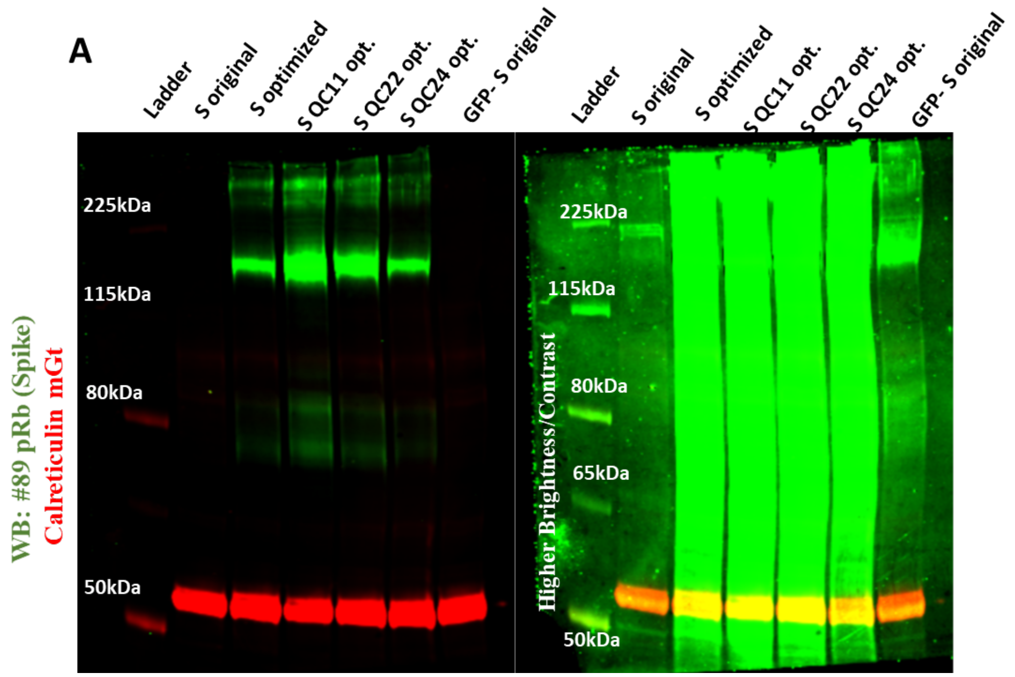

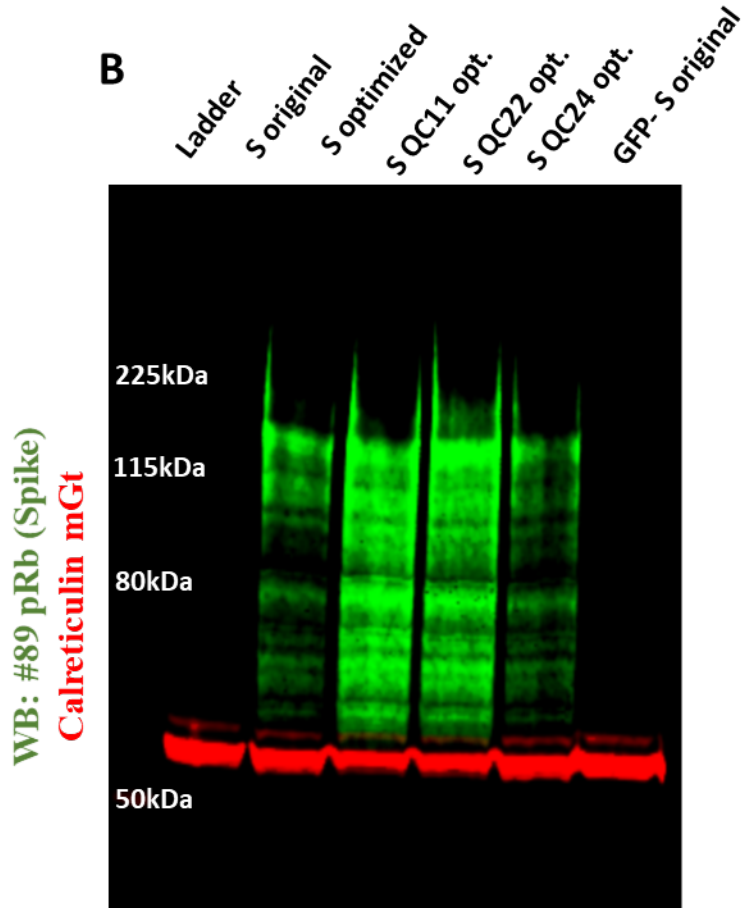

Figure S3. Lentiviral pseudovirion titers for WPRE (upper) and LTR (lower) quantitated by qPCR. Error bars indicate the standard deviation.

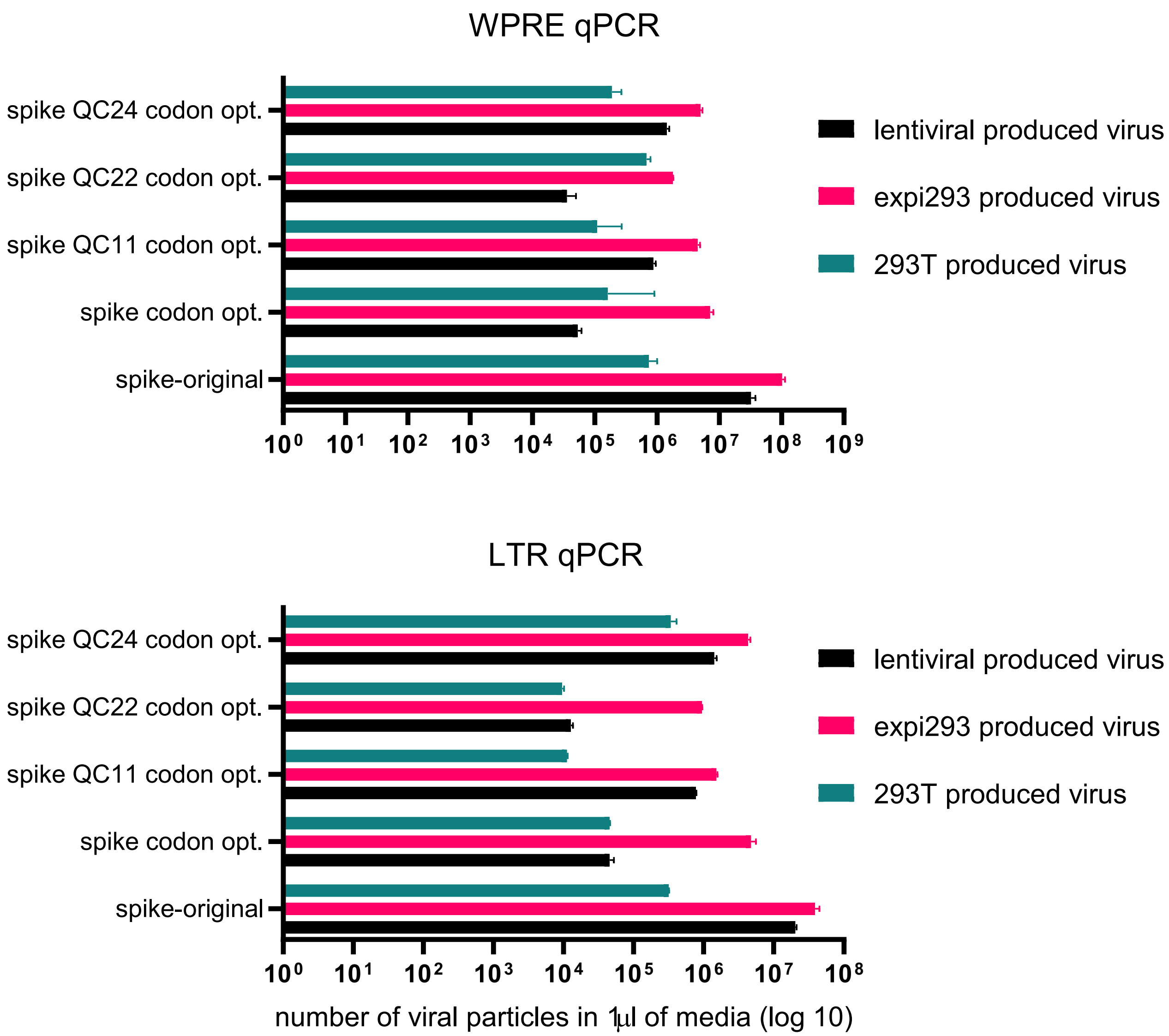

Figure S4. ARPE-19 cell infection with Expi293 produced lentiviral pseudovirions. Experiments were repeated several times. Scale bar=180 µm.

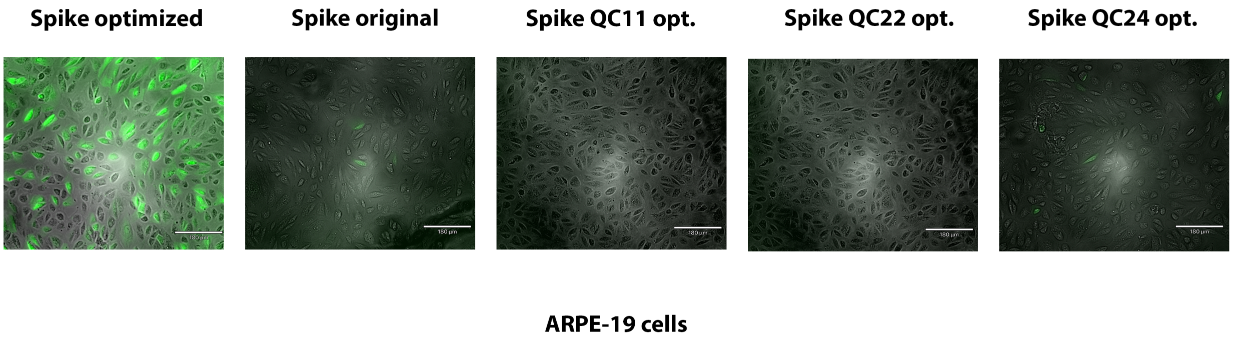

Figure S5. Vero E6 infection with Expi293-produced pseudovirions. Experiments were repeated several times. Scale bar=180 µm.

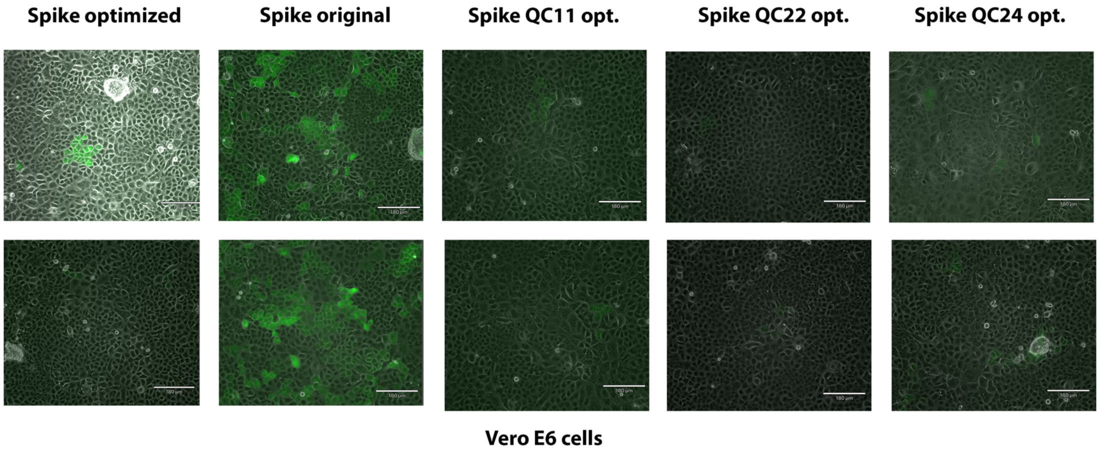

Table S1. Codon usage in protein-coding genes of viral and human genomes. Codons (CCT, CGG, and GCA) featuring in the -PRRA- insert are highlighted in yellow. CAT and CTT codons are highlighted in blue. CG-containing codons are highlighted in green. CDS = protein-coding sequences.

Codon	SARS-CoV2 CDS	SARS CoV-2 CDS,%	Human CDS	Human CDS, %
AAA	2448	6.63	993621	2.45
AAC	280	0.76	776603	1.92
AAG	1097	2.97	1295568	3.20
AAT	2873	7.78	689701	1.70
ACA	1458	3.95	614523	1.52
ACC	54	0.15	768147	1.90
ACG	63	0.17	246105	0.61
ACT	632	1.71	533609	1.32
AGA	602	1.63	494682	1.22
AGC	47	0.13	791383	1.95
AGG	47	0.13	486463	1.20
AGT	478	1.3	493429	1.22
ATA	1106	3.	304565	0.75
ATC	191	0.52	846466	2.09
ATG	762	2.06	896005	2.21
ATT	1955	5.3	650473	1.61
CAA	625	1.69	501911	1.24
CAC	29	0.08	613713	1.51
CAG	298	0.81	1391973	3.43
CAT	576	1.56	441711	1.09
CCA	476	1.29	688038	1.70
CCC	11	0.03	804620	1.99
CCG	48	0.13	281570	0.69
CCT	446	1.21	713233	1.76
CGA	120	0.33	250760	0.62
CGC	29	0.08	423516	1.05
CGG	9	0.02	464485	1.15
CGT	316	0.86	184609	0.46
CTA	75	0.2	290751	0.72
CTC	49	0.13	796638	1.97
CTG	29	0.08	1611801	3.98
CTT	780	2.11	536515	1.32
GAA	2049	5.55	1177632	2.91
GAC	233	0.63	1020595	2.52
GAG	380	1.03	1609975	3.97
GAT	2750	7.45	885429	2.18
GCA	1026	2.78	643471	1.59
GCC	28	0.08	1127679	2.78
GCG	76	0.21	299495	0.74
GCT	452	1.22	750096	1.85
GGA	537	1.46	669873	1.65
GGC	68	0.18	903565	2.23
GGG	31	0.08	669768	1.65
GGT	986	2.67	437126	1.08
GTA	844	2.29	287712	0.71
GTC	53	0.14	588138	1.45
GTG	79	0.21	1143534	2.82
GTT	923	2.5	448607	1.11
TAC	134	0.36	622407	1.54
TAT	1998	5.41	495699	1.22
TCA	1010	2.74	496448	1.22
TCC	51	0.14	718892	1.77
TCG	37	0.1	179419	0.44
TCT	853	2.31	618711	1.53
TGC	69	0.19	513028	1.27
TGG	342	0.93	535595	1.32
TGT	485	1.31	430311	1.06
TTA	1468	3.98	311881	0.77
TTC	332	0.9	824692	2.03
TTG	374	1.01	525688	1.30
TTT	1229	3.33	714298	1.76

Table S2. Statistics of the CCT-CGG-CGG-GCA stretch of codons and other sequences encoding -PRRA- in sense and antisense strands of human protein-coding sequences. All three reading frames of the antisense strand were analyzed. Human protein-coding sequences were obtained from the CCDS database (release 22, ftp.ncbi.nlm.nih.gov/pub/CCDS/). The SARS-CoV-2 S protein RNA -CCTCGGCGGGCA- insert is indicated in red.

Stretch of codons	Sense	Antisense
CCCAGGAGGGCC	4	40
CCCCGCCGGGCC	8	34
CCCCGCAGGGCC	2	31
CCCCGCAGGGCT	0	30
**CCTCGGCGGGCA**	**0**	**29**
CCCAGGAGGGCA	2	27
CCCAGAAGAGCA	5	26
CCCCGCCGCGCC	6	26
CCTCGAAGAGCT	0	24
CCACGAAGAGCT	0	23
CCCCGGAGAGCC	2	23
CCCCGGCGGGCA	5	23
CCCAGAAGAGCT	0	22
CCTCGGAGAGCA	0	21
CCCCGCAGGGCA	0	21
CCCAGAAGGGCT	1	21
CCGCGCCGCGCG	7	21
CCGCGCCGGGCC	7	21
CCCAGGAGAGCA	1	20
CCGCGCCGCGCC	7	20
CCCAGAAGGGCA	0	19
CCACGCCGTGCT	1	19
CCAAGAAGAGCC	5	19
CCTCGAAGAGCA	0	18
CCCAGAAGAGCC	3	18
CCTCGGAGGGCA	5	18
CCACGTAGGGCT	0	17
CCTCGTAGAGCT	0	17
CCCAGGAGGGCG	2	17
CCACGGCGGGCA	0	16
CCCCGCCGGGCA	2	16
CCCAGGAGAGCC	2	16
CCCAGACGGGCC	3	16
CCGAGGAGGGCT	0	15
CCACGAAGAGCA	0	15
CCTCGAAGGGCC	0	15
CCTCGCAGAGCC	0	15
CCTCGGCGGGCG	1	15
CCCAGGAGGGCT	2	15
CCTCGGCGCGCA	0	14
CCACGAAGAGCC	0	14
CCTCGCAGGGCT	0	14
CCACGGCGGGCT	0	14
CCACGCCGGGCG	1	14
CCCCGCCGGGCT	1	14

CCTCGGAGGGCC	1	14
CCGCGGCGCGCT	2	14
CCCAGGCGGGCC	3	14
CCTCGTAGGGCG	0	13
CCGAGAAGAGCA	0	13
CCTCGGCGTGCT	0	13
CCACGGAGGGCG	0	13
CCACGCCGGGCA	0	13
CCGCGCCGGGCG	1	13
CCACGAAGGGCA	1	13
CCTCGGAGAGCT	1	13
CCACGCAGGGCC	1	13
CCGCGGCGCGCA	2	13
CCCAGGCGGGCA	4	13
CCGCGGCGTGCA	0	12
CCGAGGCGGGCG	0	12
CCTCGTAGAGCG	0	12
CCTCGAAGAGCG	0	12
CCACGTCGGGCA	0	12
CCTCGGCGCGCG	0	12
CCTCGGCGCGCT	0	12
CCCCGCAGAGCA	0	12
CCACGGAGGGCT	0	12
CCCCGGCGGGCT	0	12
CCGAGGAGGGCA	1	12
CCAAGAAGGGCA	1	12
CCTCGGAGGGCT	1	12
CCAAGAAGAGCT	1	12
CCGCGGAGGGCC	2	12
CCGAGGAGGGCC	2	12
CCTCGCCGGGCA	2	12
CCGAGGAGAGCA	3	12
CCACGTAGAGCA	0	11
CCGAGGCGGGCA	0	11
CCTCGTCGCGCT	0	11
CCTCGGAGGGCG	0	11
CCTCGAAGGGCT	0	11
CCACGGCGCGCA	0	11
CCACGAAGGGCC	0	11
CCTCGCAGAGCT	0	11
CCCAGGAGAGCT	0	11
CCGAGGCGAGCC	1	11
CCCCGAAGAGCT	1	11
CCCCGGCGCGCA	1	11
CCCCGGAGGGCA	1	11
CCCCGGCGCGCC	1	11
CCACGCAGGGCA	2	11
CCGAGGCGGGCT	3	11
CCCCGGAGAGCA	4	11
CCACGCAGAGCG	7	11
CCACGTCGTGCA	0	10
CCGCGTCGCGCT	0	10
CCGCGCCGAGCA	0	10

CCACGAAGAGCG	0	10
CCGCGTCGGGCC	0	10
CCGCGCCGCGCA	0	10
CCACGGCGTGCA	0	10
CCTCGTAGAGCA	0	10
CCACGTCGGGCT	0	10
CCACGTAGAGCT	0	10
CCGAGGAGAGCT	0	10
CCCCGACGGGCA	0	10
CCTCGGCGAGCT	0	10
CCCCGACGCGCT	0	10
CCGCGCAGAGCC	0	10
CCGCGGCGGGCC	0	10
CCCCGACGGGCT	0	10
CCACGGAGAGCT	0	10
CCCCGGCGGGCC	0	10
CCACGGAGAGCA	1	10
CCACGCAGGGCT	1	10
CCACGCCGGGCC	1	10
CCCAGGCGGGCG	2	10
CCAAGAAGAGCA	3	10
CCCCGGAGGGCC	4	10
CCGCGGCGGGCG	5	10
CCGCGGCGCGCG	6	10
CCCCGCCGGGCG	6	10
CCGCGCCGTGCA	0	9
CCACGCCGTGCG	0	9
CCTCGTCGGGCA	0	9
CCTCGCAGGGCG	0	9
CCTCGTAGGGCT	0	9
CCCCGAAGGGCA	0	9
CCACGTAGGGCC	0	9
CCACGCCGCGCA	0	9
CCACGCCGGGCT	0	9
CCAAGACGGGCA	0	9
CCCAGACGGGCA	0	9
CCTCGCCGGGCC	0	9
CCACGCAGAGCC	0	9
CCCCGCAGAGCC	0	9
CCGCGCCGGGCA	1	9
CCGCGCCGCGCT	1	9
CCGCGCCGGGCT	1	9
CCGCGGCGCGCC	1	9
CCAAGGCGGGCA	1	9
CCTCGGCGGGCT	1	9
CCACGGCGGGCC	1	9
CCGCGGCGGGCA	2	9
CCTCGCCGGGCG	2	9
CCGCGCAGGGCC	2	9
CCGCGCCGAGCC	3	9
CCCCGCCGAGCC	3	9
CCAAGGAGGGCA	3	9
CCCCGCAGAGCT	3	9

CCGCGCAGGGCG	4	9
CCGAGGCGCGCG	0	8
CCTCGTCGGGCG	0	8
CCACGTAGGGCA	0	8
CCCCGCCGTGCT	0	8
CCACGACGCGCT	0	8
CCCCGAAGAGCA	0	8
CCTCGCCGCGCA	0	8
CCCCGCCGCGCT	0	8
CCAAGGAGAGCT	0	8
CCCAGGCGCGCC	0	8
CCCAGAAGGGCC	0	8
CCGAGAAGAGCT	1	8
CCCCGGCGGGCG	1	8
CCGAGGCGGGCC	1	8
CCAAGGAGAGCC	1	8
CCCCGACGGGCC	2	8
CCCCGCCGCGCG	3	8
CCACGGAGAGCC	11	8
CCTCGCCGTGCG	0	7
CCGAGAAGGGCG	0	7
CCGCGGCGAGCA	0	7
CCACGACGAGCA	0	7
CCTCGTCGCGCA	0	7
CCACGGCGTGCT	0	7
CCTCGCAGAGCG	0	7
CCACGACGCGCA	0	7
CCGAGAAGGGCT	0	7
CCCCGCAGAGCG	0	7
CCCCGTAGAGCT	0	7
CCCCGCCGAGCT	0	7
CCCCGAAGGGCT	0	7
CCCAGGCGTGCC	0	7
CCACGCCGCGCT	0	7
CCTCGCCGCGCT	0	7
CCTCGAAGAGCC	0	7
CCAAGACGGGCC	0	7
CCTAGGAGAGCC	0	7
CCGCGGAGGGCG	1	7
CCTCGCCGCGCG	1	7
CCTCGTCGGGCT	1	7
CCCCGCAGGGCG	1	7
CCGAGAAGGGCC	1	7
CCTCGCAGGGCC	1	7
CCACGGAGGGCC	1	7
CCTCGGAGAGCC	2	7
CCAAGGAGGGCC	4	7
CCGCGTAGGGCA	0	6
CCACGTCGGGCG	0	6
CCACGGCGTGCG	0	6
CCACGTAGAGCG	0	6
CCGAGGAGGGCG	0	6
CCCCGTCGTGCT	0	6

CCGAGAAGAGCG	0	6
CCACGACGCGCG	0	6
CCGCGAAGGGCT	0	6
CCGCGAAGAGCT	0	6
CCACGACGAGCT	0	6
CCCCGTCGAGCC	0	6
CCGAGACGGGCT	0	6
CCGCGGAGAGCT	0	6
CCTCGTCGCGCC	0	6
CCAAGGCGAGCT	0	6
CCAAGGCGTGCC	0	6
CCTCGTAGGGCC	0	6
CCGAGGAGAGCC	0	6
CCAAGGCGCGCA	0	6
CCTCGCAGAGCA	0	6
CCACGGAGGGCA	0	6
CCTAGGAGGGCA	0	6
CCCCGGCGCGCG	1	6
CCTCGTCGGGCC	1	6
CCCAGACGGGCT	1	6
CCACGTCGAGCT	2	6
CCACGCCGTGCC	2	6
CCCCGGCGAGCT	2	6
CCCCGAAGGGCC	2	6
CCCCGGAGAGCT	2	6
CCGCGCAGGGCT	3	6
CCCAGGCGAGCA	3	6
CCAAGGAGAGCA	4	6
CCAAGAAGGGCT	9	6
CCGCGTCGCGCG	0	5
CCGCGTCGCGCA	0	5
CCGCGCAGAGCG	0	5
CCCAGGCGTGCG	0	5
CCACGGCGAGCG	0	5
CCTCGACGTGCT	0	5
CCTCGAAGGGCG	0	5
CCCCGACGGGCG	0	5
CCCCGAAGGGCG	0	5
CCGCGAAGGGCC	0	5
CCGAGAAGGGCA	0	5
CCCCGGCGTGCA	0	5
CCAAGACGTGCT	0	5
CCCCGTAGGGCT	0	5
CCCCGGAGAGCG	0	5
CCACGAAGGGCT	0	5
CCACGTCGGGCC	0	5
CCACGTAGAGCC	0	5
CCCAGGCGAGCT	0	5
CCCAGGCGCGCA	0	5
CCAAGGCGGGCT	0	5
CCCCGGCGCGCT	0	5
CCTCGGCGCGCC	0	5
CCAAGGCGGGCC	0	5

CCAAGGCGGGCG	1	5
CCAAGAAGAGCG	1	5
CCCCGGAGGGCT	1	5
CCAAGAAGGGCC	1	5
CCCAGGCGTGCT	2	5
CCTAGGAGAGCA	2	5
CCACGCCGCGCC	2	5
CCCCGGAGGGCG	3	5
CCGCGCCGTGCG	0	4
CCGCGTCGGGCG	0	4
CCGCGGCGTGCG	0	4
CCGAGACGTGCA	0	4
CCACGTCGTGCT	0	4
CCTCGTCGTGCT	0	4
CCACGTAGGGCG	0	4
CCGCGAAGGGCA	0	4
CCTCGACGGGCG	0	4
CCACGAAGGGCG	0	4
CCCCGAAGAGCG	0	4
CCTCGCCGTGCA	0	4
CCGCGGCGAGCC	0	4
CCGCGACGGGCC	0	4
CCCCGACGAGCT	0	4
CCCCGCCGTGCA	0	4
CCCCGTCGCGCA	0	4
CCGCGGAGAGCA	0	4
CCCAGACGTGCA	0	4
CCTCGGAGAGCG	0	4
CCCCGTCGGGCT	0	4
CCTCGAAGGGCA	0	4
CCCCGCCGTGCC	0	4
CCGCGGAGAGCC	0	4
CCGAGAAGAGCC	0	4
CCTCGCAGGGCA	0	4
CCTAGAAGGGCA	0	4
CCCAGGCGCGCT	0	4
CCCAGGCGGGCT	0	4
CCCCGCCGAGCA	1	4
CCCAGAAGAGCG	1	4
CCACGTCGCGCC	1	4
CCCCGGCGTGCC	2	4
CCCAGACGCGCA	2	4
CCCCGAAGAGCC	2	4
CCTAGGAGAGCT	2	4
CCTCGGCGGGCC	2	4
CCCCGCCGCGCA	3	4
CCGCGCCGTGCC	4	4
CCTCGTCGAGCT	4	4
CCTCGCCGAGCT	4	4
CCTCGCCGGGCT	6	4
CCGCGAAGAGCG	0	3
CCTCGTCGTGCA	0	3
CCCCGACGAGCG	0	3

CCGCGGCGTGCT	0	3
CCGCGTCGGGCT	0	3
CCGCGTAGGGCT	0	3
CCGCGCCGAGCT	0	3
CCTCGTCGTGCC	0	3
CCGCGTAGGGCC	0	3
CCGCGACGCGCC	0	3
CCACGTCGCGCA	0	3
CCTCGACGTGCC	0	3
CCGCGCAGAGCA	0	3
CCTCGGCGTGCA	0	3
CCACGTCGCGCT	0	3
CCACGCAGGGCG	0	3
CCGAGACGCGCT	0	3
CCCCGTCGCGCT	0	3
CCCCGTAGAGCA	0	3
CCACGCCGAGCA	0	3
CCAAGGAGGGCG	0	3
CCCAGGCGCGCG	0	3
CCCAGACGCGCG	0	3
CCTCGCCGTGCC	0	3
CCAAGACGAGCT	0	3
CCTCGCCGAGCC	0	3
CCACGCAGAGCA	0	3
CCTAGGCGCGCA	0	3
CCACGGCGCGCT	0	3
CCCAGACGCGCC	0	3
CCTCGCCGAGCG	1	3
CCCCGGCGAGCG	1	3
CCACGGCGGGCG	1	3
CCACGGCGTGCC	1	3
CCTCGGCGTGCC	1	3
CCCCGTCGGGCC	1	3
CCTAGGCGGGCC	1	3
CCGCGACGCGCT	2	3
CCCAGGAGAGCG	2	3
CCCCGGCGAGCA	2	3
CCGAGGCGCGCC	2	3
CCTCGTCGAGCG	3	3
CCCCGGCGTGCT	3	3
CCGCGTCGTGCG	0	2
CCGCGTCGTGCA	0	2
CCACGTCGTGCG	0	2
CCGAGGCGTGCG	0	2
CCGCGACGTGCA	0	2
CCGCGACGCGCG	0	2
CCTCGACGTGCG	0	2
CCGAGACGAGCG	0	2
CCGCGTCGTGCC	0	2
CCGCGACGAGCA	0	2
CCACGTCGCGCG	0	2
CCGCGCCGTGCT	0	2
CCTCGTCGCGCG	0	2

CCCCGTCGTGCA	0	2
CCGCGTAGAGCT	0	2
CCGAGACGGGCG	0	2
CCGCGACGAGCC	0	2
CCACGTCGAGCA	0	2
CCGAGGCGAGCA	0	2
CCGCGACGGGCT	0	2
CCACGTCGTGCC	0	2
CCCCGACGAGCA	0	2
CCACGCCGTGCA	0	2
CCACGACGTGCC	0	2
CCGCGCAGGGCA	0	2
CCAAGGCGTGCA	0	2
CCGCGAAGAGCC	0	2
CCACGGCGCGCG	0	2
CCTCGTAGGGCA	0	2
CCGCGCAGAGCT	0	2
CCCCGTAGGGCA	0	2
CCTAGGCGGGCG	0	2
CCAAGGCGAGCA	0	2
CCCAGACGTGCT	0	2
CCACGCCGAGCT	0	2
CCACGGCGAGCT	0	2
CCCCGTCGCGCC	0	2
CCGAGACGGGCC	0	2
CCTCGTAGAGCC	0	2
CCCCGTAGGGCC	0	2
CCCCGTAGAGCC	0	2
CCACGACGCGCC	0	2
CCTCGACGCGCC	0	2
CCACGACGGGCC	0	2
CCACGGCGAGCC	0	2
CCTAGACGGGCA	0	2
CCCAGGCGAGCC	0	2
CCAAGGAGGGCT	0	2
CCTAGAAGGGCT	0	2
CCACGGCGCGCC	0	2
CCGCGGCGGGCT	1	2
CCACGCCGAGCC	1	2
CCTCGACGGGCC	1	2
CCCCGGCGAGCC	2	2
CCTAGAAGAGCA	2	2
CCTCGCCGAGCA	3	2
CCTAGGAGGGCC	3	2
CCAAGGCGAGCC	4	2
CCTCGACGGGCT	5	2
CCTCGCCGCGCC	5	2
CCGCGTCGTGCT	0	1
CCGCGTAGGGCG	0	1
CCGAGACGTGCG	0	1
CCGCGTCGAGCA	0	1
CCGCGGCGAGCG	0	1
CCGCGAAGGGCG	0	1

CCCCGACGTGCG	0	1
CCGAGGCGTGCA	0	1
CCTCGGCGTGCG	0	1
CCGAGGCGTGCT	0	1
CCCCGTCGCGCG	0	1
CCAAGACGTGCG	0	1
CCGAGACGTGCT	0	1
CCTAGGCGTGCG	0	1
CCGAGGAGAGCG	0	1
CCCCGTCGGGCG	0	1
CCTCGTCGAGCA	0	1
CCCCGTAGGGCG	0	1
CCCAGACGTGCG	0	1
CCCCGTAGAGCG	0	1
CCACGCCGAGCG	0	1
CCGCGAAGAGCA	0	1
CCCCGTCGAGCA	0	1
CCCCGACGTGCA	0	1
CCGCGTCGCGCC	0	1
CCGCGGCGAGCT	0	1
CCCCGACGCGCG	0	1
CCCCGCCGAGCG	0	1
CCCCGTCGAGCT	0	1
CCCCGACGTGCT	0	1
CCTCGACGAGCA	0	1
CCCAGGCGAGCG	0	1
CCTCGACGAGCT	0	1
CCACGTCGAGCC	0	1
CCGAGACGAGCC	0	1
CCGAGGCGCGCT	0	1
CCCCGTCGGGCA	0	1
CCAAGGCGTGCT	0	1
CCCAGGCGTGCA	0	1
CCGCGGAGGGCT	0	1
CCACGGAGAGCG	0	1
CCTAGACGTGCT	0	1
CCAAGAAGGGCG	0	1
CCTCGACGAGCC	0	1
CCTAGGAGGGCG	0	1
CCACGGCGAGCA	0	1
CCACGACGGGCA	0	1
CCTAGAAGGGCG	0	1
CCTCGACGGGCA	0	1
CCCCGACGAGCC	0	1
CCCAGAAGGGCG	0	1
CCTAGGCGAGCT	0	1
CCGAGACGCGCC	0	1
CCACGCAGAGCT	0	1
CCTAGGCGCGCT	0	1
CCCAGACGAGCC	0	1
CCAAGGCGCGCC	0	1
CCAAGACGCGCC	0	1
CCTAGAAGGGCC	0	1

CCACGACGGGCG	1	1
CCTAGAAGAGCG	1	1
CCTCGGCGAGCA	1	1
CCTAGGCGAGCC	1	1
CCGCGCCGAGCG	2	1
CCACGACGTGCT	2	1
CCTCGCCGTGCT	3	1
CCGCGACGTGCC	1	0
CCGCGACGGGCA	1	0
CCCAGACGAGCG	1	0
CCCCGTCGTGCC	1	0
CCACGCCGCGCG	1	0
CCAAGACGTGCC	1	0
CCTAGGAGGGCT	1	0
CCCAGACGTGCC	2	0
CCAAGACGAGCC	2	0

Table S3. Peptide identification highlighted on sequence coverage maps for different S protein products. Analysis by liquid chromatography-tandem mass spectrometry of peptides from in-gel trypsinization. A, Codon-optimized S protein (full size); B, QC24 (S2 fragment); C, QC11 (S2 fragment); D, QC24 full S protein; E, QC11 full S protein; and F, QC22 full S protein.

A. Codon-optimized S protein (full size)

Accession	Description	False Discovery Rate
A0A087WVQ6	Clathrin heavy chain OS=Homo sapiens OX=9606 GN=CLTC PE=1 SV=1	0
Q86UP2	Kinectin OS=Homo sapiens OX=9606 GN=KTN1 PE=1 SV=1	0
Q9NYU2	UDP-glucose:glycoprotein glucosyltransferase 1 OS=Homo sapiens OX=9606 GN=UGGT1 PE=1 SV=3	0
Q15075	Early endosome antigen 1 OS=Homo sapiens OX=9606 GN=EEA1 PE=1 SV=2	0
P0DTC2	Spike glycoprotein (Full Length) OS=Severe acute respiratory syndrome coronavirus 2 OX=2697049 GN=S PE=1 SV=1	0
P07814	Bifunctional glutamate/proline--tRNA ligase OS=Homo sapiens OX=9606 GN=EPRS PE=1 SV=5	0
Q14152	Eukaryotic translation initiation factor 3 subunit A OS=Homo sapiens OX=9606 GN=EIF3A PE=1 SV=1	0
Q13576	Ras GTPase-activating-like protein IQGAP2 OS=Homo sapiens OX=9606 GN=IQGAP2 PE=1 SV=4	0
Q9NTJ3	Structural maintenance of chromosomes protein 4 OS=Homo sapiens OX=9606 GN=SMC4 PE=1 SV=2	0

MFVFLVLLPLVSSQCVNLTTRTQLPPAYTNSFTRGVYYPDKVFRSSVLHSTQDLFLPFFSNVTWFHAIHVSGTNGTKRFD

NPVLPFNDGVYFASTEKSNIIRGWIFGTTLDSKTQSLLIVNNATNVVIKVCEFQFCNDPFLGVYYHKNNKSWMESEFRVY

SSANNCTFEYVSQPFLMDLEGKQGNFKNLREFVFKNIDGYFKIYSKHTPINLVRDLPQGFSALEPLVDLPIGINITRFQT

LLALHRSYLTPGDSSSGWTAGAAAYYVGYLQPRTFLLKYNENGTITDAVDCALDPLSETKCTLKSFTVEKGIYQTSNFRV

QPTESIVRFPNITNLCPFGEVFNATRFASVYAWNRKRISNCVADYSVLYNSASFSTFKCYGVSPTKLNDLCFTNVYADSF

VIRGDEVRQIAPGQTGKIADYNYKLPDDFTGCVIAWNSNNLDSKVGGNYNYLYRLFRKSNLKPFERDISTEIYQAGSTPC

NGVEGFNCYFPLQSYGFQPTNGVGYQPYRVVVLSFELLHAPATVCGPKKSTNLVKNKCVNFNFNGLTGTGVLTESNKKFL

PFQQFGRDIADTTDAVRDPQTLEILDITPCSFGGVSVITPGTNTSNQVAVLYQDVNCTEVPVAIHADQLTPTWRVYSTGS

NVFQTRAGCLIGAEHVNNSYECDIPIGAGICASYQTQTNSPRRARSVASQSIIAYTMSLGAENSVAYSNNSIAIPTNFTI

SVTTEILPVSMTKTSVDCTMYICGDSTECSNLLLQYGSFCTQLNRALTGIAVEQDKNTQEVFAQVKQIYKTPPIKDFGGF

NFSQILPDPSKPSKRSFIEDLLFNKVTLADAGFIKQYGDCLGDIAARDLICAQKFNGLTVLPPLLTDEMIAQYTSALLAG

TITSGWTFGAGAALQIPFAMQMAYRFNGIGVTQNVLYENQKLIANQFNSAIGKIQDSLSSTASALGKLQDVVNQNAQALN

TLVKQLSSNFGAISSVLNDILSRLDKVEAEVQIDRLITGRLQSLQTYVTQQLIRAAEIRASANLAATKMSECVLGQSKRV

DFCGKGYHLMSFPQSAPHGVVFLHVTYVPAQEKNFTTAPAICHDGKAHFPREGVFVSNGTHWFVTQRNFYEPQIITTDNT

FVSGNCDVVIGIVNNTVYDPLQPELDSFKEELDKYFKNHTSPDVDLGDISGINASVVNIQKEIDRLNEVAKNLNESLIDL

QELGKYEQYIKWPWYIWLGFIAGLIAIVMVTIMLCCMTSCCSCLKGCCSCGSCCKFDEDDSEPVLKGVKLHYT

413/1273*100 =32% coverage

B. QC24 (S2 fragment)

Prohibitin OS=Homo sapiens OX=9606 GN=PHB PE=1 SV=1	0
Eukaryotic translation initiation factor 3 subunit B OS=Homo sapiens OX=9606 GN=EIF3B PE=1 SV=3	0
Bifunctional glutamate/proline--tRNA ligase OS=Homo sapiens OX=9606 GN=EPRS PE=1 SV=5	0
RNA-binding protein 26 OS=Homo sapiens OX=9606 GN=RBM26 PE=1 SV=3	0
Drebrin OS=Homo sapiens OX=9606 GN=DBN1 PE=1 SV=4	0
Interleukin enhancer-binding factor 2 OS=Homo sapiens OX=9606 GN=ILF2 PE=1 SV=1	0
Spike glycoprotein (C-terminal Fragment) OS=Severe acute respiratory syndrome coronavirus 2 OX=2697049 GN=S PE=1 SV=1	0

MFVFLVLLPLVSSQCVNLTTRTQLPPAYTNSFTRGVYYPDKVFRSSVLHSTQDLFLPFFSNVTWFHAIHVSGTNGTKRFD

NPVLPFNDGVYFASTEKSNIIRGWIFGTTLDSKTQSLLIVNNATNVVIKVCEFQFCNDPFLGVYYHKNNKSWMESEFRVY

SSANNCTFEYVSQPFLMDLEGKQGNFKNLREFVFKNIDGYFKIYSKHTPINLVRDLPQGFSALEPLVDLPIGINITRFQT

LLALHRSYLTPGDSSSGWTAGAAAYYVGYLQPRTFLLKYNENGTITDAVDCALDPLSETKCTLKSFTVEKGIYQTSNFRV

QPTESIVRFPNITNLCPFGEVFNATRFASVYAWNRKRISNCVADYSVLYNSASFSTFKCYGVSPTKLNDLCFTNVYADSF

VIRGDEVRQIAPGQTGKIADYNYKLPDDFTGCVIAWNSNNLDSKVGGNYNYLYRLFRKSNLKPFERDISTEIYQAGSTPC

NGVEGFNCYFPLQSYGFQPTNGVGYQPYRVVVLSFELLHAPATVCGPKKSTNLVKNKCVNFNFNGLTGTGVLTESNKKFL

PFQQFGRDIADTTDAVRDPQTLEILDITPCSFGGVSVITPGTNTSNQVAVLYQDVNCTEVPVAIHADQLTPTWRVYSTGS

NVFQTRAGCLIGAEHVNNSYECDIPIGAGICASYQTQTNSPRRARSVASQSIIAYTMSLGAENSVAYSNNSIAIPTNFTI

SVTTEILPVSMTKTSVDCTMYICGDSTECSNLLLQYGSFCTQLNRALTGIAVEQDKNTQEVFAQVKQIYKTPPIKDFGGF

NFSQILPDPSKPSKRSFIEDLLFNKVTLADAGFIKQYGDCLGDIAARDLICAQKFNGLTVLPPLLTDEMIAQYTSALLAG

TITSGWTFGAGAALQIPFAMQMAYRFNGIGVTQNVLYENQKLIANQFNSAIGKIQDSLSSTASALGKLQDVVNQNAQALN

TLVKQLSSNFGAISSVLNDILSRLDKVEAEVQIDRLITGRLQSLQTYVTQQLIRAAEIRASANLAATKMSECVLGQSKRV

DFCGKGYHLMSFPQSAPHGVVFLHVTYVPAQEKNFTTAPAICHDGKAHFPREGVFVSNGTHWFVTQRNFYEPQIITTDNT

FVSGNCDVVIGIVNNTVYDPLQPELDSFKEELDKYFKNHTSPDVDLGDISGINASVVNIQKEIDRLNEVAKNLNESLIDL

QELGKYEQYIKWPWYIWLGFIAGLIAIVMVTIMLCCMTSCCSCLKGCCSCGSCCKFDEDDSEPVLKGVKLHYT

C. QC11 (S2 fragment)

Q16643	Drebrin OS=Homo sapiens OX=9606 GN=DBN1 PE=1 SV=4	0
P25205	DNA replication licensing factor MCM3 OS=Homo sapiens OX=9606 GN=MCM3 PE=1 SV=3	0
E9PLK3	Aminopeptidase OS=Homo sapiens OX=9606 GN=NPEPPS PE=1 SV=1	0
P35221	Catenin alpha-1 OS=Homo sapiens OX=9606 GN=CTNNA1 PE=1 SV=1	0
A0A0A0MT26	Sodium/potassium-transporting ATPase subunit alpha-3 OS=Homo sapiens OX=9606 GN=ATP1A3 PE=1 SV=1	0
P0DTC2	Spike glycoprotein (Predominantly C-terminal Fragment) OS=Severe acute respiratory syndrome coronavirus 2 OX=2697049 GN=S PE=1 S	0

MFVFLVLLPLVSSQCVNLTTRTQLPPAYTNSFTRGVYYPDKVFRSSVLHSTQDLFLPFFSNVTWFHAIHVSGTNGTKRFD

NPVLPFNDGVYFASTEKSNIIRGWIFGTTLDSKTQSLLIVNNATNVVIKVCEFQFCNDPFLGVYYHKNNKSWMESEFRVY

SSANNCTFEYVSQPFLMDLEGKQGNFKNLREFVFKNIDGYFKIYSKHTPINLVRDLPQGFSALEPLVDLPIGINITRFQT

LLALHRSYLTPGDSSSGWTAGAAAYYVGYLQPRTFLLKYNENGTITDAVDCALDPLSETKCTLKSFTVEKGIYQTSNFRV

QPTESIVRFPNITNLCPFGEVFNATRFASVYAWNRKRISNCVADYSVLYNSASFSTFKCYGVSPTKLNDLCFTNVYADSF

VIRGDEVRQIAPGQTGKIADYNYKLPDDFTGCVIAWNSNNLDSKVGGNYNYLYRLFRKSNLKPFERDISTEIYQAGSTPC

NGVEGFNCYFPLQSYGFQPTNGVGYQPYRVVVLSFELLHAPATVCGPKKSTNLVKNKCVNFNFNGLTGTGVLTESNKKFL

PFQQFGRDIADTTDAVRDPQTLEILDITPCSFGGVSVITPGTNTSNQVAVLYQDVNCTEVPVAIHADQLTPTWRVYSTGS

NVFQTRAGCLIGAEHVNNSYECDIPIGAGICASYQTQTNSPRRARSVASQSIIAYTMSLGAENSVAYSNNSIAIPTNFTI

SVTTEILPVSMTKTSVDCTMYICGDSTECSNLLLQYGSFCTQLNRALTGIAVEQDKNTQEVFAQVKQIYKTPPIKDFGGF

NFSQILPDPSKPSKRSFIEDLLFNKVTLADAGFIKQYGDCLGDIAARDLICAQKFNGLTVLPPLLTDEMIAQYTSALLAG

TITSGWTFGAGAALQIPFAMQMAYRFNGIGVTQNVLYENQKLIANQFNSAIGKIQDSLSSTASALGKLQDVVNQNAQALN

TLVKQLSSNFGAISSVLNDILSRLDKVEAEVQIDRLITGRLQSLQTYVTQQLIRAAEIRASANLAATKMSECVLGQSKRV

DFCGKGYHLMSFPQSAPHGVVFLHVTYVPAQEKNFTTAPAICHDGKAHFPREGVFVSNGTHWFVTQRNFYEPQIITTDNT

FVSGNCDVVIGIVNNTVYDPLQPELDSFKEELDKYFKNHTSPDVDLGDISGINASVVNIQKEIDRLNEVAKNLNESLIDL

QELGKYEQYIKWPWYIWLGFIAGLIAIVMVTIMLCCMTSCCSCLKGCCSCGSCCKFDEDDSEPVLKGVKLHYT

D. QC24 full S protein

P07814	Bifunctional glutamate/proline--tRNA ligase OS=Homo sapiens OX=9606 GN=EPRS PE=1 SV=5	0
P39060	Collagen alpha-1(XVIII) chain OS=Homo sapiens OX=9606 GN=COL18A1 PE=1 SV=5	0
P13645	Keratin, type I cytoskeletal 10 OS=Homo sapiens OX=9606 GN=KRT10 PE=1 SV=6	0
O60841	Eukaryotic translation initiation factor 5B OS=Homo sapiens OX=9606 GN=EIF5B PE=1 SV=4	0
Q9NTJ3	Structural maintenance of chromosomes protein 4 OS=Homo sapiens OX=9606 GN=SMC4 PE=1 SV=2	0
P0DTC2	Spike glycoprotein (Full Length) OS=Severe acute respiratory syndrome coronavirus 2 OX=2697049 GN=S PE=1 SV=1	0

MFVFLVLLPLVSSQCVNLTTRTQLPPAYTNSFTRGVYYPDKVFRSSVLHSTQDLFLPFFSNVTWFHAIHVSGTNGTKRFD

NPVLPFNDGVYFASTEKSNIIRGWIFGTTLDSKTQSLLIVNNATNVVIKVCEFQFCNDPFLGVYYHKNNKSWMESEFRVY

SSANNCTFEYVSQPFLMDLEGKQGNFKNLREFVFKNIDGYFKIYSKHTPINLVRDLPQGFSALEPLVDLPIGINITRFQT

LLALHRSYLTPGDSSSGWTAGAAAYYVGYLQPRTFLLKYNENGTITDAVDCALDPLSETKCTLKSFTVEKGIYQTSNFRV

QPTESIVRFPNITNLCPFGEVFNATRFASVYAWNRKRISNCVADYSVLYNSASFSTFKCYGVSPTKLNDLCFTNVYADSF

VIRGDEVRQIAPGQTGKIADYNYKLPDDFTGCVIAWNSNNLDSKVGGNYNYLYRLFRKSNLKPFERDISTEIYQAGSTPC

NGVEGFNCYFPLQSYGFQPTNGVGYQPYRVVVLSFELLHAPATVCGPKKSTNLVKNKCVNFNFNGLTGTGVLTESNKKFL

PFQQFGRDIADTTDAVRDPQTLEILDITPCSFGGVSVITPGTNTSNQVAVLYQDVNCTEVPVAIHADQLTPTWRVYSTGS

NVFQTRAGCLIGAEHVNNSYECDIPIGAGICASYQTQTNSPRRARSVASQSIIAYTMSLGAENSVAYSNNSIAIPTNFTI

SVTTEILPVSMTKTSVDCTMYICGDSTECSNLLLQYGSFCTQLNRALTGIAVEQDKNTQEVFAQVKQIYKTPPIKDFGGF

NFSQILPDPSKPSKRSFIEDLLFNKVTLADAGFIKQYGDCLGDIAARDLICAQKFNGLTVLPPLLTDEMIAQYTSALLAG

TITSGWTFGAGAALQIPFAMQMAYRFNGIGVTQNVLYENQKLIANQFNSAIGKIQDSLSSTASALGKLQDVVNQNAQALN

TLVKQLSSNFGAISSVLNDILSRLDKVEAEVQIDRLITGRLQSLQTYVTQQLIRAAEIRASANLAATKMSECVLGQSKRV

DFCGKGYHLMSFPQSAPHGVVFLHVTYVPAQEKNFTTAPAICHDGKAHFPREGVFVSNGTHWFVTQRNFYEPQIITTDNT

FVSGNCDVVIGIVNNTVYDPLQPELDSFKEELDKYFKNHTSPDVDLGDISGINASVVNIQKEIDRLNEVAKNLNESLIDL

QELGKYEQYIKWPWYIWLGFIAGLIAIVMVTIMLCCMTSCCSCLKGCCSCGSCCKFDEDDSEPVLKGVKLHYT

28% coverage

E. QC11 full S protein

A0A087WVQ6	Clathrin heavy chain OS=Homo sapiens OX=9606 GN=CLTC PE=1 SV=1
Q86UP2	Kinectin OS=Homo sapiens OX=9606 GN=KTN1 PE=1 SV=1
Q15075	Early endosome antigen 1 OS=Homo sapiens OX=9606 GN=EEA1 PE=1 SV=2
Q9NYU2	UDP-glucose:glycoprotein glucosyltransferase 1 OS=Homo sapiens OX=9606 GN=UGGT1 PE=1 SV=3
P0DTC2	Spike glycoprotein OS=Severe acute respiratory syndrome coronavirus 2 OX=2697049 GN=S PE=1 SV=1

MFVFLVLLPLVSSQCVNLTTRTQLPPAYTNSFTRGVYYPDKVFRSSVLHSTQDLFLPFFSNVTWFHAIHVSGTNGTKRFD

NPVLPFNDGVYFASTEKSNIIRGWIFGTTLDSKTQSLLIVNNATNVVIKVCEFQFCNDPFLGVYYHKNNKSWMESEFRVY

SSANNCTFEYVSQPFLMDLEGKQGNFKNLREFVFKNIDGYFKIYSKHTPINLVRDLPQGFSALEPLVDLPIGINITRFQT

LLALHRSYLTPGDSSSGWTAGAAAYYVGYLQPRTFLLKYNENGTITDAVDCALDPLSETKCTLKSFTVEKGIYQTSNFRV

QPTESIVRFPNITNLCPFGEVFNATRFASVYAWNRKRISNCVADYSVLYNSASFSTFKCYGVSPTKLNDLCFTNVYADSF

VIRGDEVRQIAPGQTGKIADYNYKLPDDFTGCVIAWNSNNLDSKVGGNYNYLYRLFRKSNLKPFERDISTEIYQAGSTPC

NGVEGFNCYFPLQSYGFQPTNGVGYQPYRVVVLSFELLHAPATVCGPKKSTNLVKNKCVNFNFNGLTGTGVLTESNKKFL

PFQQFGRDIADTTDAVRDPQTLEILDITPCSFGGVSVITPGTNTSNQVAVLYQDVNCTEVPVAIHADQLTPTWRVYSTGS

NVFQTRAGCLIGAEHVNNSYECDIPIGAGICASYQTQTNSPRRARSVASQSIIAYTMSLGAENSVAYSNNSIAIPTNFTI

SVTTEILPVSMTKTSVDCTMYICGDSTECSNLLLQYGSFCTQLNRALTGIAVEQDKNTQEVFAQVKQIYKTPPIKDFGGF

NFSQILPDPSKPSKRSFIEDLLFNKVTLADAGFIKQYGDCLGDIAARDLICAQKFNGLTVLPPLLTDEMIAQYTSALLAG

TITSGWTFGAGAALQIPFAMQMAYRFNGIGVTQNVLYENQKLIANQFNSAIGKIQDSLSSTASALGKLQDVVNQNAQALN

TLVKQLSSNFGAISSVLNDILSRLDKVEAEVQIDRLITGRLQSLQTYVTQQLIRAAEIRASANLAATKMSECVLGQSKRV

DFCGKGYHLMSFPQSAPHGVVFLHVTYVPAQEKNFTTAPAICHDGKAHFPREGVFVSNGTHWFVTQRNFYEPQIITTDNT

FVSGNCDVVIGIVNNTVYDPLQPELDSFKEELDKYFKNHTSPDVDLGDISGINASVVNIQKEIDRLNEVAKNLNESLIDL

QELGKYEQYIKWPWYIWLGFIAGLIAIVMVTIMLCCMTSCCSCLKGCCSCGSCCKFDEDDSEPVLKGVKLHYT

463/1273*100=36% coverage

F. QC22 full S protein

Q14152	Eukaryotic translation initiation factor 3 subunit A OS=Homo sapiens OX=9606 GN=EIF3A PE=1 SV=1
Q9NTJ3	Structural maintenance of chromosomes protein 4 OS=Homo sapiens OX=9606 GN=SMC4 PE=1 SV=2
O60841	Eukaryotic translation initiation factor 5B OS=Homo sapiens OX=9606 GN=EIF5B PE=1 SV=4
P39060	Collagen alpha-1(XVIII) chain OS=Homo sapiens OX=9606 GN=COL18A1 PE=1 SV=5
Q6P2E9	Enhancer of mRNA-decapping protein 4 OS=Homo sapiens OX=9606 GN=EDC4 PE=1 SV=1
P0DTC2	Spike glycoprotein OS=Severe acute respiratory syndrome coronavirus 2 OX=2697049 GN=S PE=1 SV=1

MFVFLVLLPLVSSQCVNLTTRTQLPPAYTNSFTRGVYYPDKVFRSSVLHSTQDLFLPFFSNVTWFHAIHVSGTNGTKRFD

NPVLPFNDGVYFASTEKSNIIRGWIFGTTLDSKTQSLLIVNNATNVVIKVCEFQFCNDPFLGVYYHKNNKSWMESEFRVY

SSANNCTFEYVSQPFLMDLEGKQGNFKNLREFVFKNIDGYFKIYSKHTPINLVRDLPQGFSALEPLVDLPIGINITRFQT

LLALHRSYLTPGDSSSGWTAGAAAYYVGYLQPRTFLLKYNENGTITDAVDCALDPLSETKCTLKSFTVEKGIYQTSNFRV

QPTESIVRFPNITNLCPFGEVFNATRFASVYAWNRKRISNCVADYSVLYNSASFSTFKCYGVSPTKLNDLCFTNVYADSF

VIRGDEVRQIAPGQTGKIADYNYKLPDDFTGCVIAWNSNNLDSKVGGNYNYLYRLFRKSNLKPFERDISTEIYQAGSTPC

NGVEGFNCYFPLQSYGFQPTNGVGYQPYRVVVLSFELLHAPATVCGPKKSTNLVKNKCVNFNFNGLTGTGVLTESNKKFL

PFQQFGRDIADTTDAVRDPQTLEILDITPCSFGGVSVITPGTNTSNQVAVLYQDVNCTEVPVAIHADQLTPTWRVYSTGS

NVFQTRAGCLIGAEHVNNSYECDIPIGAGICASYQTQTNSPRRARSVASQSIIAYTMSLGAENSVAYSNNSIAIPTNFTI

SVTTEILPVSMTKTSVDCTMYICGDSTECSNLLLQYGSFCTQLNRALTGIAVEQDKNTQEVFAQVKQIYKTPPIKDFGGF

NFSQILPDPSKPSKRSFIEDLLFNKVTLADAGFIKQYGDCLGDIAARDLICAQKFNGLTVLPPLLTDEMIAQYTSALLAG

TITSGWTFGAGAALQIPFAMQMAYRFNGIGVTQNVLYENQKLIANQFNSAIGKIQDSLSSTASALGKLQDVVNQNAQALN

TLVKQLSSNFGAISSVLNDILSRLDKVEAEVQIDRLITGRLQSLQTYVTQQLIRAAEIRASANLAATKMSECVLGQSKRV

DFCGKGYHLMSFPQSAPHGVVFLHVTYVPAQEKNFTTAPAICHDGKAHFPREGVFVSNGTHWFVTQRNFYEPQIITTDNT

FVSGNCDVVIGIVNNTVYDPLQPELDSFKEELDKYFKNHTSPDVDLGDISGINASVVNIQKEIDRLNEVAKNLNESLIDL

QELGKYEQYIKWPWYIWLGFIAGLIAIVMVTIMLCCMTSCCSCLKGCCSCGSCCKFDEDDSEPVLKGVKLHYT

331/1273*100=26% coverage

Table S4. Expression and infectivity of pseudovirions produced in different cells. WPRE qPCR, LTR qPCR, p24 and %GFP positive Vero E6 infected cells with lentiviral pseudovirions containing S proteins produced in LV (lenti), Expi293 (expi) and HEK293T cells. The data are presented as means ± S.D. of 3 measurements.

qPCR WPRE particle/μl	LV			Expi293			HEK293T		
Spike original	3.24E+07	±	5.52E+06	1.02E+08	±	1.15E+07	7.43E+05	±	2.62E+05
Spike codon opt.	5.31E+04	±	8.44E+03	7.13E+06	±	8.79E+05	1.61E+05	±	7.43E+05
Spike QC11 codon opt.	8.67E+05	±	8.51E+04	4.48E+06	±	4.11E+05	1.09E+05	±	1.61E+05
Spike QC22 codon opt.	3.56E+04	±	1.46E+04	1.81E+06	±	1.31E+04	6.76E+05	±	1.09E+05
Spike QC24 codon opt.	1.44E+06	±	1.32E+05	5.02E+06	±	3.90E+05	1.88E+05	±	8.18E+04

qPCR LTR particle/μl	LV			Expi293			HEK293T		
Spike original	2.02E+07	±	9.57E+05	3.85E+07	±	6.29E+06	3.165E+05	±	2.84E+03
Spike codon opt.	4.51E+04	±	7.35E+03	4.74E+06	±	8.41E+05	4.538E+04	±	5.81E+02
Spike QC11 codon opt.	7.74E+05	±	1.24E+04	1.51E+06	±	5.70E+04	1.118E+04	±	2.94E+02
Spike QC22 codon opt.	1.27E+04	±	9.76E+02	9.41E+05	±	1.56E+04	9.441E+03	±	7.52E+02
Spike QC24 codon opt.	1.41E+06	±	1.18E+05	4.29E+06	±	3.66E+05	3.39E+05	±	6.87E+04

%GFP at 48h post-infection	LV			Expi293			HEK293T		
Spike original	95.21	±	0.28	58.69	±	1.2	34.36	±	2.8
Spike codon opt.	0.01	±	0.0014	1.97	±	0.5	10.34	±	0.44
Spike QC11 codon opt.	0.61	±	0.24	1.19	±	0.26	15.78	±	0.53
Spike QC22 codon opt.	0.01	±	0.006	1.06	±	0.09	24.78	±	3
Spike QC24 codon opt.	1.45	±	0.01	1.47	±	0.04	20.3	±	0.14

p24 ng/ml	LV	Expi293	HEK293T
Spike original	16631	11262	4137
Spike codon opt.	172	2202	2005
Spike QC11 codon opt.	257	1594	668
Spike QC22 codon opt.	50	700	1820
Spike QC24 codon opt.	237	2832	164
